# Prevalence, outcomes, and cost of chronic kidney disease in a contemporary population of 2·4 million patients from 11 countries: The CaReMe CKD study

**DOI:** 10.1016/j.lanepe.2022.100438

**Published:** 2022-06-30

**Authors:** Johan Sundström, Johan Bodegard, Andreas Bollmann, Marc G. Vervloet, Patrick B. Mark, Avraham Karasik, Tiago Taveira-Gomes, Manuel Botana, Kåre I. Birkeland, Marcus Thuresson, Levy Jäger, Manish M. Sood, Gijs VanPottelbergh, Navdeep Tangri, CaReMe CKD Investigators

**Affiliations:** a Department of Medical Sciences, Uppsala University, Uppsala, Sweden; b The George Institute for Global Health, University of New South Wales, Sydney, Australia; c Cardiovascular, Renal and Metabolism, Medical Department, BioPharmaceuticals, AstraZeneca, Oslo, Norway; d Heart Center Leipzig at University of Leipzig and Leipzig Heart Institute, Leipzig, Germany; e Amsterdam UMC, Department of Nephrology, Amsterdam, the Netherlands; f Institute of Cardiovascular and Medical Sciences, University of Glasgow, Glasgow, UK; g Maccabi Institute for Research and Innovation, Maccabi Healthcare Services, Tel Aviv, Israel; h Department of Community Medicine, Information and Decision in Health, Faculty of Medicine, University of Porto, Portugal; i University Hospital Lucus Augusti, Lugo, Spain; j Department of transplantation medicine, Oslo University Hospital, Oslo, Norway; k University of Oslo, Oslo, Norway; l Statisticon AB, Uppsala, Sweden; m Institute of Primary Care, University of Zurich and University Hospital Zurich, Zurich, Switzerland; n Ottawa Hospital Research Institute, University of Ottawa, Ottawa, Ontario, Canada; o Department of Public Health and Primary Care, KUleuven, Belgium; p Department of Medicine and Community Health Sciences, University of Manitoba, Winnipeg, Canada

**Keywords:** Chronic kidney disease, Renal impairment, Prevalence, Epidemiology, Outcomes, Costs, Primary care, Public health, Global health

## Abstract

**Background:**

Digital healthcare systems data could provide insights into the global prevalence of chronic kidney disease (CKD). We designed the CaReMe CKD study to estimate the prevalence, key clinical adverse outcomes and costs of CKD across 11 countries.

**Methods:**

Individual-level data of a cohort of 2·4 million contemporaneous CKD patients was obtained from digital healthcare systems in participating countries using a pre-specified common protocol; summarized using random effects meta-analysis. CKD and its stages were defined in accordance with current Kidney Disease: Improving Global Outcomes (KDIGO) criteria. CKD was defined by laboratory values or by a diagnosis code.

**Findings:**

The pooled prevalence of possible CKD was 10·0% (95% confidence interval 8.5‒11.4; mean pooled age 75, 53% women, 38% diabetes, 60% using renin-angiotensin-aldosterone system inhibitors). Two out of three CKD patients identified by laboratory criteria did not have a corresponding CKD-specific diagnostic code. Among CKD patients identified by laboratory values, the majority (42%) were in KDIGO stage 3A; and this fraction was fairly consistent across countries. The share with CKD based on urine albumin-creatinine ratio (UACR) alone (KDIGO stages one and two) was 29%, with a substantial heterogeneity between countries. Adverse events were common; 6·5% were hospitalized for CKD or heart failure, and 6·2% died, annually. Costs for renal events and heart failure were consistently higher than costs for atherosclerotic events in CKD patients across all countries.

**Interpretation:**

We estimate that CKD is present in one out of ten adults. These individuals experience significant adverse outcomes with associated costs. The prevalence of CKD is underestimated when using diagnostic codes alone. There is considerable public health potential in diagnosing CKD and providing treatments to those currently undiagnosed.

**Funding:**

The study was sponsored by AstraZeneca.

**Research in context**

**Evidence before the study**

To gain information on the current global prevalence and burden of chronic kidney disease (CKD), we conducted a systematic search of MEDLINE and EMBASE for studies published in the ten years preceding October 5^th^ 2021; using the search terms “chronic kidney disease OR CKD (Title)”, connected using the Boolean Operator “AND” with “prevalence OR burden OR cost (Title)”. Studies were limited to those published in the English language and to those conducted in humans. After duplicates were removed, 557 studies were read. Two relevant studies had provided estimates of the global prevalence of CKD. However, given that estimates varied widely between those two studies (9·1% *versus* 13·4%), there was a clear need to more precisely determine the prevalence of CKD and its stages in the population, as well as the corresponding burdens to patients and societies.

**Added value of the study**

To determine the prevalence of each stage of CKD and to detail patient characteristics, risks and costs associated with CKD across the participating countries, individual-level data from 2·4 million contemporaneous CKD patients across 11 countries were obtained from digital healthcare systems using a pre-specified common protocol. The available data indicates that one in ten adults in Europe, Canada and Israel likely have CKD. Of those, two out of three have not been diagnosed with CKD, and many are not treated using renin-angiotensin aldosterone system inhibitors. Costs for renal events and heart failure were consistently higher than costs for atherosclerotic events in CKD patients across all countries.

**Implications of all the available evidence**

There is considerable public health potential in diagnosing CKD using widely available low-cost testing and providing treatments to those currently undiagnosed.

Alt-text: Unlabelled box

## Introduction

Recent data suggests that 9·1% to 13·4% of the worldwide population (between 700 million and one billion people) has chronic kidney disease (CKD).^1^^,^^2^ As the global population ages, a stable age-standardized prevalence over the last three decades has led to a substantial increase in all-age prevalence of this important chronic condition.^1^ The growing burden of CKD is illustrated by its increasing contribution to total mortality and its associated financial costs,^1^^,^^3^^,^^4^ with CKD-related costs in Europe estimated with a large uncertainty at 1·3% of total healthcare costs.^6^

The prevalence estimates above may represent the true underlying distributions in different populations, but the large range of those estimates, paired with the high and increasing population health burden posed by CKD, urges for attempts to more precisely determine the prevalence of CKD and its stages in the population, as well as the corresponding burdens to patients and societies. The CaReMe CKD study was designed to address this issue; collecting detailed contemporaneous primary data from healthcare systems of 11 nations to determine the prevalence of each stage of CKD and to detail patient characteristics, risks and costs associated with CKD across the participating countries.

## Methods

### Study setting and data sources

**Figure 1:**
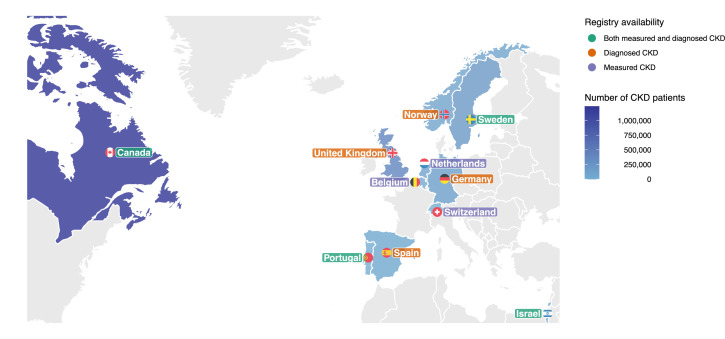
Countries and number of chronic kidney disease (CKD) patients. Number of patients with both measured and diagnosed CKD*,* defined as having a CKD diagnosis or one pathological UACR or eGFR value, where the chronicity of CKD was not confirmed. *Measured CKD*, patients with Kidney Disease: Improving Global Outcomes (KDIGO) criteria CKD using urine albumin-to-creatinine ration and estimated glomerular filtration rate. *Diagnosed CKD*, patients who have registered CKD diagnosis, with or without available pathological eGFR and/or UACR values.

**Figure 2:**
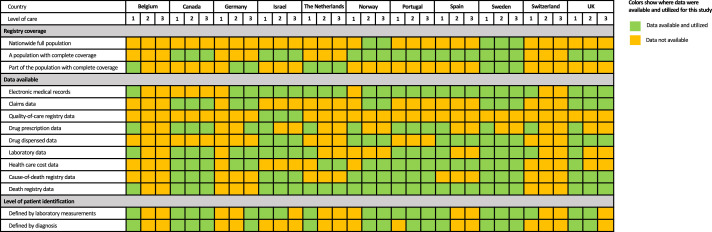
Description of data sources used across the countries. Data extractions on the following levels of health care: 1. Primary health care. 2. Secondary health care (specialist or outpatient hospital care). 3. Tertiary health care (in-hospital care).

The CaReMe (CArdioRenal and MEtabolic) study utilizes the unique features of available health care registries and corresponding health care systems’ primary data across 11 countries: Belgium, Canada, Germany, Israel, The Netherlands, Norway, Portugal, Spain, Sweden, Switzerland, and the United Kingdom (Figure 1). A detailed description of the separate data sources is available in the Supplementary Material (page 4-8). A heat map, describing the coverage of the registries, data availability and health care level for CKD identification is illustrated in Figure 2. A single pre-specified protocol was used to collect all the data in all participating countries. Background populations were estimated based on the coverage of the health care registries for countries in which this information was available. Data on estimated glomerular filtration rate (eGFR), urine albumin-creatinine ratio (UACR), and other characteristics relied on values calculated in the original data sources using the routines of those settings. Permissions from ethics authorities in each participating country was obtained before start of the study.

### Study sample

Patients were identified in any available health care registry (Figure 2), by either having a CKD diagnosis or a single pathological value of eGFR <60 ml/min/1·73 m^2^ or UACR ≥30 mg/g (≥3 mg/mmol) (Table S1).

Due to variance in sampling frames and in the availability of data between countries, three different CKD definitions were initially investigated: 1) *Possible CKD* (defined as having a CKD diagnosis or one pathological UACR or eGFR value, where the chronicity of CKD was not confirmed); 2) *Measured CKD* (two pathological UACR or eGFR values at least 90 days apart); and 3) *Diagnosed CKD* (a registered CKD diagnosis, with or without available pathological eGFR and/or UACR values) (Table S2). Other definitions were also explored: e.g., CKD identification by one pathological UACR or eGFR value (*Single-measure CKD*), two pathological UACR or eGFR values at least 90 days apart and within the last two years (*Time-limited CKD*), and by not allowing for any normalization of any UACR or eGFR value between two measurements (*Persistent CKD*) (Table S2).^5^

### Analysis groups based on CKD definitions

Analyses were performed in two groups based on how CKD could be defined in the different data sources: *Measured CKD* and *Diagnosed CKD*, as they are expected to differ in several regards.

### Patient cohorts for prevalence, event-rates and hospital health care costs

Three separate cohorts were defined in each country with a fixed index date (January 1^st^ for each index year) to describe the following: *Cohort One*, to describe the most contemporary patient characteristics; *Cohort Two*, to describe 1-year event rates; and *Cohort Three*, to describe up to five-year hospital care costs (Table S3). The index years for these three cohorts were determined separately for each country, depending on how updated the registries were. For the description of the most contemporary population (*Cohort One*), patients were indexed on the January 1^st^ in the most recent available year in each country: Belgium, 2020; Canada, 2019; Germany, 2019; Israel, 2020; Norway, 2020 Portugal, 2018; Spain, 2019; Sweden, 2019; Switzerland, 2020; and the United Kingdom, 2019) (Table S3). One-year event rates were calculated from January 1^st^ at the most recent index date, minus one year (*Cohort Two*); and hospital health care costs were calculated from the most recent index year, minus five years (*Cohort Three*), in each country.

### Baseline characteristics

For *Cohort One*, comorbidities were searched in all available data prior to the index date. There were exceptions for severe hypoglycaemia, which was searched for during the one-year period prior to the index date; and cancer, which was searched for during the five-year period prior to the index date (Table S4). Additionally, the use of drug treatment was searched for during the one-year period prior to the index date. The baseline period for laboratory data was defined as the last value collected during the three years prior to the index date.

### Clinical outcomes

For *Cohort Two*, cardiovascular and renal disease were defined by the following outcomes: CKD, heart failure (HF), stroke, myocardial infarction and peripheral artery disease, as the main diagnosis.^6^ In all countries, the first event registered between index (baseline) and the end of follow-up or death were defined by the first recorded out- or in-hospital diagnosis of CKD (including diabetic kidney disease, acute kidney failure, CKD, unspecified kidney disease, hypertensive kidney failure and dialysis), HF (including hypertensive HF), stroke (including ischemic and hemorrhagic stroke), myocardial infarction and peripheral artery disease (Table S5). Cardiorenal disease (collectively a diagnosis of HF or CKD as first diagnosis) was described as a separate entity, in addition to HF and CKD separately. If the first hospitalization was due to more than one of the diagnoses defined above, the diagnosis with highest importance (i.e., main diagnosis was considered more important than secondary diagnoses) was defined as the first cardiorenal event.

### Hospital health care costs for cardiorenal events

Hospital health care costs were calculated per country for descriptive purposes. For *Cohort Three*, hospital health care costs were extracted from data containing the actual visit costs as charged by the health care provider (e.g., the cost reflects the true reimbursement claim to the local payer). These hospital health care costs were cumulatively summarized from index and, importantly, includes costs for all first and repeated events, during follow-up, associated with the outcomes described above. Hospital health care cost data was available in Canada, Portugal, Spain and UK.

### Statistical analysis

All statistical analyses were performed in each country separately according to prespecified statistical analysis plans, using a reference statistical analysis plan from the Swedish context in terms of definition and analyses, and adapted to potential local needs regarding specific register-based details. Baseline characteristics were described using standard statistical measures, such as mean and standard deviations for numerical variables and frequencies and percentages for categorical variables. Missingness was described for key variables; complete-case analyses were used. The CKD populations are described separately by country and DerSimonian and Laird random effects meta-analysis was used when pooling data, taking heterogeneity between countries into consideration.^7^ Tau (Ʈ) was used to describe the heterogeneity; it corresponds to the estimated standard deviation of the underlying data across countries. To assess heterogeneity between *Measured* and *Diagnosed CKD* produced by differences in data availability, we performed a sensitivity analysis with only the five countries that could contribute to all estimates (Canada, Israel, The Netherlands, Portugal, and Sweden). All analyses were conducted using R statistical software (R version 3.5.0).^8^

### Event rates

Event rates were calculated as events per 100 patient-years based on time to first event, and patients were censored at death, upon leaving the database, or after one year. All analyses of event rates are descriptive, and no formal between-group comparisons have been made.

### Hospital health care costs

Hospital health care costs were first summarized annually within patient as the total cost per year per diagnosis; and then summarized within country as the mean cost per patient per year. Hospital health care costs were censored from death onwards, whereas patients leaving the database were not included in the denominator from the year after leaving the database. Results are presented separately for each country and no formal comparisons between countries have been made. All diagnoses were analysed independently from other diagnoses and, thus, events containing more than one of the targeted diagnoses contribute hospital health care costs to each of the included diagnoses. Therefore, one cannot add the hospital health care costs of two diagnoses to form a combined cost. Conversion rates was updated on the 1^st^ of January 2021 to $1 US Dollars from Canadian Dollar, Euro, Swedish Krona and British pound sterling were 0·77, 1·13, 8.56 and 1·37 respectively.

### Role of the funding source

AstraZeneca, J.B., supported the design, interpretation of results, writing of the manuscript and publication of this study together with the investigators. Study management and data extraction was coordinated by AstraZeneca in all countries. The authors were not precluded from accessing data in the study, and all authors took final responsibility in the decision to submit for publication.

## Results

Across 11 countries, 2·4 million *Possible CKD* patients were identified (Table S1). In countries with available background population estimates, the prevalence of *Possible CKD* (a CKD diagnosis or one pathological UACR or eGFR value) was 10·0%, (Table 1). The prevalence of *Measured CKD* (two pathological UACR or eGFR values at least 90 days apart) was 7·0%, of which one in three were defined by UACR and two in three were defined by eGFR. Additionally, the prevalence of *Single-measure CKD* (one pathological UACR or eGFR value), *Time-limited CKD* (two pathological UACR or eGFR values at least 90 days apart and within the last two years) and *Persistent CKD* (not allowing for normalization of any UACR or eGFR value between two) were, 9·0% (7·6‒10·4), 5·3% (3·0‒7·5) and 5·6% (3·4‒7·8) respectively (Table S6). The prevalence of CKD as defined by a diagnostic code was 3·5% (Table 1). A sensitivity analysis in the five countries that had data on both *Measured* and *Diagnosed CKD* (Tables S8-S10 and Figure S1) produced similar estimates as the main analyses; the prevalence of *Measured CKD* in this subsample was 7·4% and *Diagnosed CKD* 2·8%.

**Table 1:** Prevalence of chronic kidney disease across nine countries and a background population of 30,253,881 adults >18 years of age.

	Belgium	Canada	Israel	the Netherlands	Norway^a^	Portugal	Spain^b^	Sweden	UK	Pooled prevalence^c^	Tau
**Prevalence**											
Possible CKD	12·0%	9·8%	n/a	8·9%	n/a	11·1%	n/a	8·3%	n/a	10·0% (8·7‒11·4)	1·54
Measured CKD	5·6%	7·0%	6·5%	n/a	n/a	9·8%	n/a	6·1%	n/a	7·0% (5·6‒8·5)	1·65
*UACR CKD (Stage I-II)*	1·9%	2·4%	2·8%	n/a	n/a	3·5%	n/a	1·4%	n/a	2·4% (1·7‒3·1)	0·79
*eGFR CKD (Stage III-V)*	3·6%	4·7%	3·7%	n/a	n/a	6·3%	n/a	4·6%	n/a	4·6% (3·6‒5·5)	1·08
Diagnosed CKD	4·4%	3·4%	2·1%	n/a	2·5%	1·8%	4·8%	3·7%	6·5%	3·7% (2·6‒4·8)	1·57
**Number of patients**											
Possible CKD, n	25,156	1,235,791	n/a	194,978	n/a	11,802	n/a	212,846	n/a		
Measured CKD, n	11,744	883,310	84,229	n/a	n/a	10,455	n/a	156,230	n/a		
Diagnosed CKD, n	9293	421,795	27,868	n/a	104,116	1932	56,435	95,575	391,618		
**Background population**	208,921	12,553,761	1,298,633	2,187,962	4,153,579	106,482	1,175,426	2,570,327	5,998,790		

CKD, chronic kidney disease. *Possible CKD,* patients with a CKD diagnosis or one pathological UACR or eGFR measurement. *Measured CKD*, patients with KDIGO confirmed CKD using UACR and eGFR. *Diagnosed CKD*, patients who have a registered CKD diagnosis.

a Patients in hospital care with nationwide coverage.

b Patients were mainly identified by diagnosis with a small part (7%) by laboratory data.

c Random effects models were used to calculate pooled values, and the heterogeneity measure Ʈ (tau) corresponds to the estimated standard deviation of the underlying data. UK, United Kingdom.

### Patient characteristics

**Figure 3:**
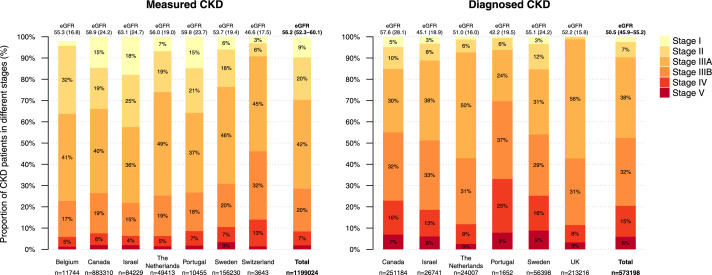
Proportion of patients in the different chronic kidney disease (CKD) stages per country in 2018 to 2021. *Measured CKD*, patients with KDIGO confirmed CKD using UACR and eGFR. *Diagnosed CKD*, patients who have a registered CKD diagnosis. Stage I: eGFR ≥90 ml/min/1·73 m^2^ and UACR 30-300 mg/g [3-30 mg/nmol] Stage II: eGFR 60-89 ml/min/1·73 m^2^ and UACR 30-300 mg/g [3-30 mg/nmol] Stage IIIA: eGFR 45-59 ml/min/1·73 m^2^ Stage IIIB: eGFR 30-44 ml/min/1·73 m^2^) Stage IV: eGFR 15-29 ml/min/1·73 m^2^ Stage V: eGFR <15 ml/min/1·73 m^2^ eGFR, estimated glomerular filtration rate, ml/min/1·73 m^2^. UACR, Urine albumin-to-creatinine ration. UK, United Kingdom.

Patients with *Measured CKD* (Table 2) and *Diagnosed CKD* (Table 3) had similar age and systolic blood pressure (<140 mmHg) profiles. However, the latter were burdened with more comorbidities, such as HF and coronary artery disease. The prevalence of diabetes was similar in both CKD categories with pooled estimates 38 and 39%. Only one in three of the *Measured CKD* patients had a CKD diagnosis. The majority of CKD patients had registered UACR measurements both in the *Measured CKD* (60%) and *Diagnosed CKD* (71%) patients. However, there were large variations in measurement rates between the countries, highest in Israel and lowest in Belgium (Tables 1 and 2). The proportion of CKD that was defined using high UACR (stage I-II) was higher in the *Measured CKD* patients (29%) than in the *Diagnosed CKD* patients (9%; Figure 3).

**Table 2:** Baseline characteristics of 1,111,836 contemporary *Measured CKD* patients defined by having KDIGO confirmed CKD.

	Belgium	Canada	Israel	the Netherlands	Portugal	Sweden	Switzerland	Pooled baseline value^a^	Tau
Number of patients, n	11,744	883,310	84,229	49,413	10,455	156,230	3802	n/a	n/a
Index year	2020	2018	2021	2019	2019	2019	2020		
Age, years (SD)	77 (12)	72 (15)	72 (13)	76 (11)	73 (14)	75 (14)	79 (11)	74·8 (72·8‒76·9)	2·73
Females, *n* (%)	7313 (62)	475,801 (54)	37,700 (45)	26,713 (54)	5471 (52)	78,486 (50)	2097 (55)	53·2 (49·3‒57·2)	5·28
CKD diagnosis, *n* (%)	4674 (40)	251,305 (28)	26,741 (32)	24,007 (49)	2054 (20)	56,398 (36)	n/a	34·1 (26·1‒42·0)	9·92
**Comorbidities**									
Heart failure, *n* (%)	1271 (11)	186,593 (21)	8682 (10)	7436 (15)	1403 (13)	37,836 (24)	n/a	15·8 (11·3‒20·4)	5·66
Coronary artery disease, *n* (%)	n/a	247,748 (28)	11,282 (13)	13,741 (28)	1075 (10)	42,537 (27)	n/a	21·4 (13·7‒29·0)	8·76
Stroke, *n* (%)	834 (7)	71,403 (8)	4424 (5)	9329 (19)	1340 (13)	29,531 (19)	n/a	11·8 (7·0‒16·6)	6·01
Atrial fibrillation/flutter, *n* (%)	2069 (18)	101,885 (12)	10,128 (12)	9345 (19)	1272 (12)	41,763 (27)	n/a	16·5 (11·8‒21·2)	5·92
Peripheral artery disease, *n* (%)	782 (7)	18,478 (2)	4621 (5)	12,496 (25)	344 (3)	11,114 (7)	n/a	8·3 (1·5‒15·1)	8·53
Diabetes, *n* (%)	3134 (27)	395,035 (45)	41,554 (49)	13,995 (28)	4885 (47)	59,667 (38)	1221 (32)	38·0 (31·2‒44·8)	9·18
Cancer, *n* (%)	3262 (28)	293,605 (33)	22,032 (26)	8052 (16)	1110 (11)	38,850 (25)	n/a	23·2 (16·6‒29·8)	8·23
**Laboratory measurements**									
Systolic blood pressure, mmHg, mean (SD)	n/a	n/a	133·9 (16·7)	138·0 (17·0)	137·5 (17·0)	137·2 (19·4)	136·3 (20·3)	136·6 (135·2‒138·0)	1·63
Sodium, mmol/L, mean (SD)	n/a	140·3 (3·2)	139·3 (2·5)	n/a	139·2 (3·1)	139·9 (3·0)	140·5 (3·1)	139·8 (139·2‒140·3)	0·57
Potassium, mmol/L, mean (SD)	4·6 (0·6)	4·4 (0·5)	4·7 (0·5)	4·3 (0·4)	4·4 (0·5)	4·5 (0·6)	4·3 (0·4)	4·5 (4·3‒4·6)	0·15
>5.5 mmol/L, *n* (%)	689 (7)	15,879 (2)	4292 (6)	358 (1)	204 (3)	5744 (5)	70 (2)	3·6 (2·0‒5·3)	2·25
Magnesium, mmol/L, mean (SD))	n/a	0·8 (0·1)	0·8 (0·1)	n/a	0·8 (0·1)	0·8 (0·2)	0·8 (0·1)	0·8 (0·8‒0·8)	0·01
Calcium, mmol/L, mean (SD)	n/a	2·3 (0·3)	2·3 (0·7)	n/a	2·3 (0·2)	n/a	2·4 (0·1)	2·3 (2·3‒2·3)	0·03
eGFR, mL/min/1.73 m2, mean (SD)	55·3 (16·8)	58·9 (24·2)	63·1 (24·7)	56·0 (19·0)	59·8 (23·7)	53·7 (19·4)	46·6 (17·5)	56·2 (52·3‒60·1)	5·27
<15	261 (2)	16,880 (2)	1641 (2)	291 (1)	184 (2)	2237 (1)	52 (1)	1·6 (1·2‒2·0)	0·53
15-29	3761 (32)	48,046 (6)	3704 (4)	2270 (5)	716 (7)	11,444 (8)	458 (12)	10·5 (3·2‒17·8)	9·84
30-44	4811 (41)	163,402 (19)	13,011 (15)	9101 (19)	1896 (18)	31,400 (21)	1170 (31)	23·6 (16·8‒30·4)	9·18
45-59	1985 (17)	341,661 (40)	30,104 (36)	23,353 (49)	3916 (37)	69,231 (46)	1627 (44)	38·4 (30·5‒46·2)	10·59
60-89	541 (5)	166,153 (19)	20,677 (25)	9360 (20)	2197 (21)	27,417 (18)	292 (8)	16·5 (11·0‒21·9)	7·33
90+	149 (1)	121,686 (14)	15,092 (18)	3211 (7)	1546 (15)	8582 (6)	123 (3)	9·1 (4·4‒13·9)	6·42
Creatinine, mg/dL, mean (SD)	n/a	1·3 (0·9)	1·2 (3·0)	1·2 (0·5)	1·2 (0·6)	1·2 (0·8)	0·1 (0·3)	1·0 (0·7‒1·4)	0·45
S-Albumin, g/dL, mean (SD)	n/a	4·0 (0·5)	4·0 (0·4)	n/a	4·0 (0·5)	n/a	4·2 (0·4)	4·0 (4·0‒4·1)	0·10
uACR, mg/g, mean (SD)	180·2 (496·4)	24·4 (73·3)	97·6 (106·7)	87·9 (322·2)	128·8 (396·6)	175·4 (546·3)	75·0 (72·3)	109·3 (68·1‒150·5)	55·29
% of patients with measurement	11·1	62·0	95·5	78·4	90·3	50·7	30·0	59·7 (36·5‒82·9)	31·35
HbA1c DCCT, %, mean (SD)	6·5 (1·0)	6·5 (1·4)	6·3 (1·3)	6·6 (1·2)	6·8 (1·4)	6·5 (1·4)	6·1 (0·8)	6·5 (6·3‒6·6)	0·22
Hemoglobin, g/dL, mean (SD)	13·2 (1·7)	13·1 (1·8)	13·3 (1·8)	13·4 (1·6)	13·2 (1·8)	13·2 (1·7)	13·1 (1·6)	13·2 (13·1‒13·3)	0·12
Hb 10-12, g/dL, *n* (%)	1983 (17)	n/a	15,187 (18)	4438 (16)	1933 (20)	30,034 (21)	779 (21)	18·8 (17·0‒20·6)	2·20
Hb ≤10, g/dL	348 (3)	n/a	2908 (3)	805 (3)	420 (4)	5627 (4)	161 (4)	3·6 (3·1‒4·1)	0·63
Hematocrit, %, mean (SD)	40·0 (16·8)	39·4 (4·9)	41·1 (5·2)	40·0 (5·0)	n/a	n/a	39·3 (4·7)	40·0 (39·3‒40·6)	0·71
<40%	5374 (47)	n/a	33,340 (40)	8580 (42)	n/a	n/a	2120 (58)	46·6 (38·6‒54·6)	8·11
**CKD treatment, *n* (%)**	5723 (49)	432,124 (49)	60,678 (72)	30,442 (62)	7356 (70)	98,666 (63)	n/a	60·8 (52·7‒68·9)	10·10
RAAS inhibitor	5199 (44)	420,463 (48)	58,735 (70)	29,321 (59)	7302 (70)	94,524 (61)	2596 (68)	59·9 (52·1‒67·7)	10·49
MRA	1134 (10)	27,332 (3)	5931 (7)	3297 (7)	685 (7)	13,628 (9)	331 (9)	7·2 (5·6‒8·8)	2·16
SGLT-2i	14 (0)	28,150 (3)	4732 (6)	287 (1)	521 (5)	3031 (2)	110 (3)	2·8 (1·2‒4·3)	2·07
Dialysis	n/a	14,837 (2)	1538 (2)	505 (1)	n/a	4394 (3)	n/a	1·8 (1·1‒2·6)	0·74

SD, Standard deviation. All numbers in parenthesis are percentage if not stated otherwise. CKD, chronic kidney disease. *Measured CKD*, patients with KDIGO confirmed CKD using UACR and eGFR. CKD, chronic kidney disease. RAAS, renin angiotensin aldosterone system. MRA, mineralocorticoid receptor antagonist. SGLT-2i, sodium-glucose-cotransporter-2-inhibitors. DCCT, Diabetes Control and Complications Trial units.

a Random effects models were used to calculate pooled values, and the heterogeneity measure Ʈ (tau) corresponds to the estimated standard deviation of the underlying data.

**Table 3:** Baseline characteristics of 1,222,526 contemporary *Diagnosed CKD* patients defined by having a registered CKD diagnosis.

	Canada	Germany	Israel	The Netherlands	Norway^a^	Portugal	Spain^b^	Sweden	UK	Pooled baseline value^c^	Tau
Number of patients, n	421,795	161,407	27,868	33,723	104,116	1932	56,435	95,575	391,618	n/a	n/a
Index year	2018	2019	2021	2019	2020	2019	2018	2019	2019		
Age, years (SD)	68 (17)	77 (11)	75 (12)	75 (12)	70 (16)	78 (11)	76 (14)	68 (19)	75 (14)	73·5 (71·0‒76·0)	3·83
Females, *n* (%)	206,645 (49)	81,193 (50)	10,427 (37)	18,672 (55)	43,074 (41)	965 (50)	26,957 (48)	46,052 (48)	225,933 (58)	48·6 (44·5‒52·6)	6·24
CKD diagnosis, *n* (%)	421,795 (100)	161,407 (100)	27,868 (100)	33,723 (100)	104,116 (100)	1932 (100)	56,435 (100)	95,575 (100)	391,618 (100)	100·0 (100·0‒100·0)	0·00
**Comorbidities**											
Heart failure, *n* (%)	113,464 (27)	63,683 (39)	4963 (18)	5365 (16)	22,734 (22)	750 (39)	11,610 (21)	21,284 (22)	65,748 (17)	24·5 (18·6‒30·3)	8·91
Coronary artery disease, *n* (%)	126,325 (30)	21,695 (13)	5032 (18)	9222 (27)	30,260 (29)	440 (23)	10,519 (19)	22,339 (23)	123,088 (31)	23·8 (19·7‒27·8)	6·18
Stroke, *n* (%)	40,780 (10)	13,328 (8)	2014 (7)	6415 (19)	3118 (3)	461 (24)	5967 (11)	15,750 (16)	62,720 (16)	12·7 (8·4‒16·9)	6·55
Atrial fibrillation/flutter, *n* (%)	62,629 (15)	51,717 (32)	4529 (16)	6410 (19)	26,853 (26)	535 (28)	8921 (16)	20,915 (22)	79,006 (20)	21·5 (17·6‒25·3)	5·90
Peripheral artery disease, *n* (%)	13,027 (3)	14,817 (9)	2112 (8)	8701 (26)	10,109 (10)	183 (9)	2700 (5)	6602 (7)	26,794 (7)	9·3 (5·0‒13·6)	6·58
Diabetes, *n* (%)	162,212 (38)	61,029 (38)	14,673 (53)	8280 (25)	29,308 (28)	1093 (57)	27,394 (49)	33,507 (35)	112,196 (29)	38·9 (31·5‒46·3)	11·36
Cancer, *n* (%)	145,254 (34)	18,694 (12)	8260 (30)	5610 (17)	29,604 (28)	389 (20)	9026 (16)	20,099 (21)	59,760 (15)	21·5 (16·4‒26·5)	7·71
**Laboratory measurements**											
Systolic blood pressure, mmHg, mean (SD)	n/a	n/a	133·4 (17·3)	137·0 (17·0)	n/a	136·9 (18·0)	137·6 (20·2)	135·1 (20·3)	132·6 (15·8)	135·4 (133·8‒137·1)	2·07
Sodium, mmol/L, mean (SD)	140·2 (3·4)	n/a	139·3 (2·6)	n/a	n/a	139·3 (3·4)	138·5 (12·6)	139·9 (3·0)	139·9 (3·5)	139·5 (139·0‒140·0)	0·60
Potassium, mmol/L, mean (SD)	4·4 (0·5)	n/a	4·8 (0·5)	4·3 (0·4)	n/a	4·5 (0·6)	4·0 (0·8)	4·5 (0·7)	4·6 (0·5)	4·4 (4·2‒4·6)	0·25
>5.5 mmol/L, *n* (%)	8927 (2)	n/a	2888 (11)	301 (1)	n/a	91 (5)	3395 (6)	4692 (8)	11,042 (3)	5·3 (2·7‒7·9)	3·52
Magnesium, mmol/L, mean (SD))	0·8 (0·1)	n/a	0·8 (0·1)	n/a	n/a	0·8 (0·1)	n/a	0·8 (0·2)	0·8 (0·5)	0·8 (0·8‒0·8)	0·01
Calcium,, mmol/L, mean (SD)	2·2 (0·3)	n/a	2·3 (1·2)	n/a	n/a	2·3 (0·2)	9·0 (0·9)	n/a	2·4 (6·5)	3·6 (1·0‒6·3)	2·99
eGFR, mL/min/1.73 m2, mean (SD)	57·6 (28·1)	n/a	45·1 (18·9)	51·0 (16·0)	n/a	42·2 (19·5)	49·8 (20·0)	55·1 (24·2)	52·2 (15·8)	50·4 (46·5‒54·4)	5·34
Creatinine, mg/dL, mean (SD)	1·5 (1·3)	n/a	1·8 (5·2)	1·3 (0·6)	n/a	1·7 (0·8)	1·3 (0·6)	1·3 (1·0)	1·3 (0·8)	1.4 (1·3‒1·6)	0·20
S-Albumin, g/dL, mean (SD)	3·9 (0·6)	n/a	4·0 (0·4)	n/a	n/a	3·9 (0·6)	n/a	n/a	4·0 (0·5)	3·9 (3·9‒4·0)	0·08
uACR, mg/g, mean (SD)	36·3 (101·9)	n/a	111·2 (122·1)	87·2 (341·3)	n/a	214·9 (635·7)	390·8 (300·0)	264·1 (707·5)	117·3 (470·1)	174·4 (83·3‒265·6)	122·89
% of patients with measurement	58·3	n/a	95·2	70·1	n/a	90·7	100·0	40.2	42·1	70·9 (52·4‒89·5)	25·03
HbA1c DCCT, %, mean (SD)	6·3 (1·4)	n/a	6·3 (1·2)	6·7 (1·1)	n/a	6·8 (1·5)	6·8 (1·2)	6·5 (1·4)	6·2 (1·2)	6·5 (6·4‒6·7)	0·23
Hemoglobin, g/dL, mean (SD)	12·7 (1·9)	n/a	12·9 (1·9)	13·3 (1·6)	n/a	12·2 (1·8)	13·4 (1·3)	13·0 (1·8)	13·1 (1·7)	12·9 (12·7‒13·2)	0·39
Hb 10-12, g/dL, *n* (%)	n/a	n/a	7203 (26)	3355 (17)	n/a	633 (34)	3941 (7)	18,478 (24)	63,259 (20)	21·1 (13·9‒28·3)	8·97
Hb ≤10, g/dL	n/a	n/a	1769 (6)	620 (3)	n/a	208 (11)	3034 (5)	4042 (5)	11,500 (4)	5·7 (3·5‒7·9)	2·74
Hematocrit, %, mean (SD)	38·6 (5·3)	n/a	39·8 (5·5)	40·0 (5·0)	n/a	n/a	40·4 (10·9)	n/a	39·9 (4·8)	39·7 (39·1‒40·3)	0·69
<40%	n/a	n/a	14,056 (51)	6290 (44)	n/a	n/a	4896 (9)	n/a	146,214 (49)	37·9 (18·6‒57·1)	19·68
**CKD treatment *n* (%)**	175,178 (42)	n/a	20,247 (73)	20,113 (60)	n/a	1221 (63)	n/a	51,472 (54)	183,483 (47)	56·3 (47·2‒65·3)	11·31
RAAS inhibitor	168,724 (40)	n/a	19,362 (69)	19,326 (57)	53,320 (51)	1217 (63)	39,046 (69)	49,220 (51)	179,043 (46)	55·9 (48·5‒63·4)	10·76
MRA	16,396 (4)	n/a	2779 (10)	2471 (7)	6212 (6)	221 (11)	3336 (6)	7052 (7)	14,549 (4)	6·9 (5·1‒8·8)	2·65
SGLT-2i	8265 (2)	n/a	1576 (6)	102 (0)	3022 (3)	80 (4)	889 (2)	1414 (1)	2947 (1)	2·3 (1·1‒3·6)	1·79
Dialysis	16,554 (4)	6368 (4)	1500 (5)	475 (1)	5672 (5)	n/a	925 (2)	5073 (5)	4011 (1)	3·5 (2·2‒4·8)	1·89

SD, Standard deviation. All numbers in parenthesis are percentage if not stated otherwise. CKD, chronic kidney disease. *Diagnosed CKD*, patients who have a registered CKD diagnosis. RAAS, renin angiotensin aldosterone system. MRA, mineralocorticoid receptor antagonist. SGLT-2i, sodium-glucose-cotransporter-2-inhibitors. DCCT, Diabetes Control and Complications Trial units.

a Patients in hospital care with nationwide coverage.

b Patients were mainly identified by diagnosis with a small part (7%) by laboratory data.

c Random effects models were used to calculate pooled values, and the heterogeneity measure Ʈ (tau) corresponds to the estimated standard deviation of the underlying data. UK, United Kingdom.

A little more than half of patients were treated with renin-angiotensin aldosterone system (RAAS) inhibitors; slightly fewer in the *Diagnosed CKD* patients (55·9%) than in the *Measured CKD* patients (59·9%) across all countries (Tables 1 and 2). There were some variations in RAAS inhibitor treatment between countries, with the highest fractions of treated observed in Israel and Portugal (70%); and the lowest in Belgium and Canada, 44% and 48%, respectively. Approximately 2% of the *Measured CKD* patients received dialysis treatment, the rate of which was two-fold higher among *Diagnosed CKD* patients. SGLT-2 inhibitors were not indicated for CKD treatment at the time of data capture, and saw little use across all countries (2·3-2·8%).

### Rates of cardiovascular events and death

Cardiorenal event rates (CKD or HF) were consistently higher than rates of atherosclerotic events (myocardial infarction or stroke) across all countries, in both the *Measured CKD* and *Diagnosed CKD* patients (Table 4). Moreover, cardiorenal event rates were approximately twice as high among *Diagnosed CKD* patients than in *Measured CKD* patients. The rates of atherosclerotic events were more similar between those groups. Rates of cardiovascular and all-cause death were 31-49% higher in *Diagnosed CKD* patients than in *Measured CKD* patients. The share of all deaths that was attributed to renal or cardiovascular causes was similar (one in two) in the *Measured CKD* patients and *Diagnosed CKD* patients; as was the share attributed to cardiovascular causes (one in three; Table 4).

**Table 4:** Numbers of events and one-year event rates per 100 patient-years in prevalent patients with chronic kidney disease (CKD).

	Canada	Germany	Israel	The Netherlands	Norway	Portugal	Spain	Sweden	UK	Pooled event rates	Tau
***Measured CKD* patients**											
Cardiorenal disease	25564 (3·4)	n/a	3721 (4·7)	738 (3·7)	n/a	437 (5·2)	n/a	21358 (15·5)	n/a	6·50 (2·05‒10·95)	5·07
CKD	9343 (1·2)	n/a	2642 (3·4)	377 (1·9)	n/a	275 (3·3)	n/a	14038 (9·9)	n/a	3·92 (0·89‒6·96)	3·46
Heart failure	17532 (2·3)	n/a	1861 (2·4)	404 (2·0)	n/a	234 (2·8)	n/a	8652 (5·9)	n/a	3·08 (1·66‒4·50)	1·62
Myocardial infarction	6475 (0·9)	n/a	544 (0·7)	350 (0·7)	n/a	50 (0·6)	n/a	2657 (1·8)	n/a	0·92 (0·49‒1·35)	0·49
Stroke	6347 (0·8)	n/a	1005 (1·3)	656 (1·3)	n/a	120 (1·4)	n/a	3683 (2·5)	n/a	1·45 (0·91‒1·99)	0·61
Renal death	1871 (0·2)	n/a	n/a	n/a	n/a	126 (1·5)	n/a	463 (0·3)	n/a	0·67 (0·00‒1·44)	0·68
Cardiovascular death	13525 (1·8)	n/a	n/a	n/a	n/a	183 (2·1)	n/a	4130 (2·7)	n/a	2·22 (1·66‒2·79)	0·49
All cause death	43131 (5·7)	n/a	3998 (5·1)	n/a	n/a	562 (6·6)	n/a	11441 (7·6)	n/a	6·23 (5·14‒7·31)	1·10
***Diagnosed CKD* patients**											
Cardiorenal disease	18056 (5·0)	16478 (10·9)	2603 (10·3)	1200 (2·3)	23286 (28·8)	217 (15·3)	n/a	17420 (21·4)	16483 (4·5)	13·44 (6·57‒20·31)	9·27
CKD	7846 (2·1)	n/a	2183 (8·6)	517 (1·0)	18055 (21·7)	159 (11·0)	n/a	13601 (16·3)	10980 (3·0)	10·14 (3·69‒16·59)	8·05
Heart failure	11323 (3·1)	12701 (8·4)	1077 (4·3)	740 (1·4)	7057 (7·8)	102 (7·0)	n/a	5000 (5·6)	6294 (1·7)	5·35 (3·43‒7·27)	2·57
Myocardial infarction	3476 (0·9)	1086 (0·7)	266 (1·1)	170 (0·8)	2036 (2·2)	12 (0·8)	n/a	1462 (1·6)	3019 (0·8)	1·17 (0·77‒1·58)	0·54
Stroke	3115 (0·8)	1487 (1·0)	405 (1·6)	279 (1·4)	469 (0·5)	32 (2·1)	n/a	1836 (2·0)	5086 (1·4)	1·32 (0·88‒1·75)	0·58
Renal death	1657 (0·4)	430 (0·3)	n/a	n/a	457 (0·5)	81 (5·4)	n/a	427 (0·5)	769 (0·2)	1·36 (0·00‒3·22)	2·11
Cardiovascular death	8374 (2·3)	1734 (1·1)	n/a	n/a	2701 (2·9)	89 (5·9)	n/a	2444 (2·7)	7508 (2·0)	2·91 (1·45‒4·37)	1·65
All cause death	27989 (7·6)	7035 (4·6)	1927 (7·6)	n/a	9071 (9·7)	247 (16·3)	6020 (12·1)	6962 (7·6)	26981 (7·2)	9·30 (6·55‒12·06)	3·70

CKD, chronic kidney disease. *Measured CKD*, patients with KDIGO confirmed CKD using UACR and eGFR. *Diagnosed CKD*, patients who have a registered CKD diagnosis. ^b^ Not included in the pooled event rates since only in-hospital visits were obtainable. ^b^ Random effects models were used to calculate pooled values, and the heterogeneity measure Ʈ (tau) corresponds to the estimated standard deviation of the underlying data. UK, United Kingdom.

### Hospital healthcare costs

**Figure 4:**
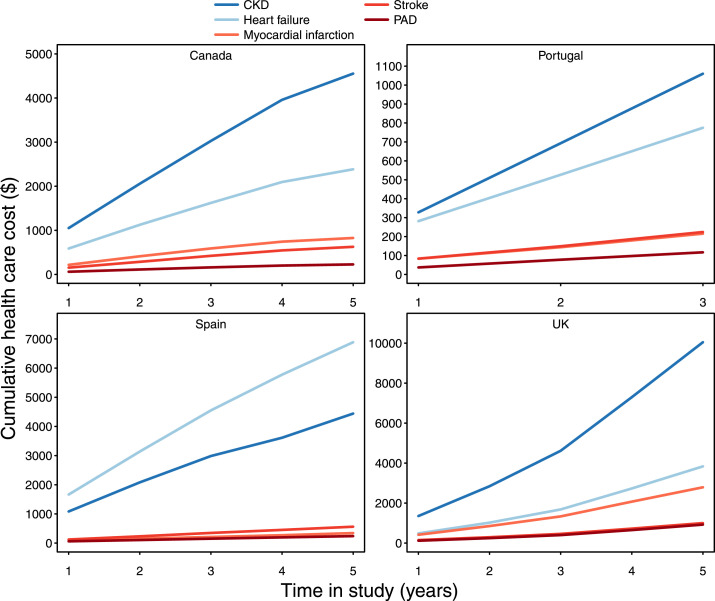
Hospital health care costs per chronic kidney disease (CKD) patient at index and cumulatively during up to 5 years. PAD, peripheral artery disease. UK, United Kingdom.

Estimates of hospital healthcare costs were available in four countries representing 1,695,704 patients; 75% of the CKD total population (Table S7). Hospital healthcare costs per CKD patient varied substantially between countries, with the highest costs observed in Spain and the lowest in Portugal. Hospital healthcare costs for cardiorenal events (CKD or HF) were consistently higher than those for atherosclerotic events (myocardial infarction or stroke), across all countries (Figure 4). The cumulative health care costs for cardiorenal events and atherosclerotic events increased similarly (three to eight times) within the countries during the five-year follow-up (Table S7).

## Discussion

In this study, we examined 2·4 million patients with CKD using primary data from digital healthcare systems in 11 countries, providing contemporary estimates of the totality of the burden of CKD in Europe, Canada and Israel. We estimated the pooled prevalence of possible CKD at 10% in adult populations; and noted that two out of three CKD patients had not been diagnosed. Mortality was substantial in this population, and the leading cause of hospital visits and costs was CKD, followed by HF.

Among the broad and deep patient characteristics available (Tables 2 and 3), we noted that CKD was equally common in men and women and that the average person with CKD is 74-75 years old, that is significantly older than in previous reports).^2^^,^^9^ Diabetes was present in 38-39% of the CKD patients, that is slightly higher than reported in previous studies.^1^^,^^2^ Further, coronary artery disease was present in 21-24%, followed by HF in 16-25%, with the higher shares in those CKD patients identified by a diagnosis code for CKD. The prevalence of these traits in the CKD population have hitherto been uncertain. Atrial fibrillation and stroke were also common among CKD patients, underscoring that the CKD population is multimorbid and requires a multifaceted approach. Further, the fractions with hyperkalemia and anemia were substantial, highlighting the attention that these traits in CKD require. We noted that a RAAS inhibitor was, on average, given to 60% of those with measures-identified CKD. However, this fraction was somewhat heterogeneous, from <50% in Belgium and Canada to 70% in Portugal and Israel. The low RAAS inhibitor use in CKD suggests that there is actionable opportunity to improve treatment and prognosis.

The rates of cardiorenal events and death among the CKD patients were substantial (Table 4). Hospitalization rates were highest for CKD events (4% per year among CKD patients identified using laboratory values, and 10% for those identified with a diagnosis code). This was closely followed by rates of HF hospitalizations (3% per year among CKD patients identified using laboratory values, and 5% for those identified with a diagnosis code). Death rates were substantial in the CKD patients as well (6% per year in CKD patients identified using laboratory values and 9% in patients identified with a diagnosis code). These rates were, to a large extent, previously unknown. Atherosclerotic cardiovascular disease event rates were much lower in this general CKD population, but is more important in end-stage kidney disease samples. Of note, hospitalizations may be captured with higher sensitivity in settings where patients were identified by healthcare and given a diagnosis code than in settings where patients were identified afterwards using laboratory values. We have therefore presented event rates for those groups separately (Table 4).

Costs of care for CKD patients are difficult to compare between countries given the differences in healthcare systems and cost accounting. However, relative patterns between cost entities are possible to rank within countries and compare. A fairly consistent picture emerged with CKD-related care as the foremost driver of costs in most countries, with HF care in second place. Atherosclerotic cardiovascular disease did not accrue healthcare costs at the level associated with CKD and HF.

The prevalence of possible CKD in the present study (10%) was relatively close to the recent Global Burden of Disease study (9·1%),^1^ albeit calculated using quite different methodology; and lower than a previous estimate that summarized secondary data (13·4%).^2^ Underestimations of the CKD prevalence may arise from the use of population-based cohort studies suffering from the healthy participant effect.^1^ Overestimations may be caused by determining the CKD diagnosis using single time-point determinations of kidney function (inconsistent with the Kidney Disease: Improving Global Outcomes [KDIGO] clinical practice guidelines designed to confirm the chronicity of kidney abnormalities)^1^^,^^10^^,^^11^ or by the selection of high-risk groups.^2^ Additionally, imprecision in estimates may arise from a lack of primary data in many countries, reliance on diagnosis codes for determining stages of CKD, and sampling error.^1^^,^^2^ It is possible that the various potential biases in this present study and the recent Global Burden of Disease study,^1^ which provide estimates of comparable magnitude, are small and that the studies do, in fact, provide reliable estimates of the underlying true prevalence of CKD. In that case, the same estimates were reached using very different analytical methods and data sources. The Global Burden of Disease study uses population-based cohorts (over-representing the healthy), and the present study used digital healthcare systems data (over-representing the unhealthy). In our study, heterogeneity in the prevalence estimates between countries was fairly low. Nonetheless, important insights can be gained by deeper scrutiny of some individual countries. For example, in Portugal, primary care programs frequently remind patients to visit their family doctor by letter at least once every three years, so laboratory values available for this study are quite representative of the entire adult population in Portugal. The prevalence of CKD in Portugal was slightly above the overall average; this could be a result of those regular assessments, or it could be due to chance or other biases.

It is notable that among those with CKD determined from laboratory measures using the KDIGO criteria (i.e., values indicating CKD present at two consecutive measurement occasions at least three months apart), only 34% had been diagnosed with CKD (Table 2). This low fraction is likely to reflect the intensity of kidney care provided. Of those with laboratory measures-identified CKD (measured CKD), the majority, 42%, were in stage IIIA; and this fraction was fairly consistent across countries (Figure 3). The fraction with CKD that was defined based on UACR (stages I and II) was 29%, with substantial heterogeneity between countries. This likely represents differences in the use of urine sampling in primary care, with a variable proportion of point-of-care dipsticks used. It is also noteworthy that in settings identifying CKD patients using a diagnosis code (e.g., ICPC, SNOMED, ICD), a very small fraction had CKD stages I and II, suggesting that awareness and diagnosis of these CKD stages remains very low (Figure 3).

Some limitations of this study are noteworthy. The data stem from sometimes very different sources, and sampling biases are likely. Nonetheless, these are some of the best available real-world data to gauge the prevalence of CKD and patient characteristics. To determine the population prevalence of CKD, the study uses healthcare data for the numerator, which underrepresent the healthy and asymptomatic that do not seek healthcare. As they are present in the denominator, this may lead to an underestimation of the prevalence of CKD. One notable exception to this differential misclassification bias may be Portugal, where patients are reminded to get health check-ups every three years, which may produce a less biased estimate. Of note, aside from screening programs, those that do show up in healthcare are a relevant population as they are the ones available for diagnosis and preventive efforts using the high-risk strategy. Further, routine health records typically lack data on race and other valuable characteristics. The generalizability of our results to populations with very different circumstances in terms of race, resources or care is unknown. Diagnosis codes are associated with some misclassification; we therefore only considered main diagnoses when assessing outcomes. Hospital health care costs vary significantly between countries due to differences in health care and reimbursement structures. In this study, we relied on administrative claims by the hospitals, with limited possibilities to break down costs into detailed in-hospital cost items. We assumed that the national health care and reimbursement structure specifics would affect different diseases similarly, and that within-country ranking of costs for different diseases would therefore be possible.^12^ Renal replacement therapy costs were handled differently in different countries, and this is likely to affect some within-country rankings; notably, rankings were nonetheless quite similar between countries. Strengths of the study include the unparalleled size of the sample of contemporaneous CKD patients, the unprecedented multinational compilation of real-world healthcare data of total populations, and the consistency of the findings across diverse countries despite differences in ethnicity, socioeconomic status, healthcare systems, and treatment guidelines. Other strengths include the validity of the CKD diagnosis used in this study,^6^^,^^13^ data collection undertaken using a pre-specified protocol, the availability of multiple varieties of clinical characteristics of the patients, and the availability of the total hospital healthcare costs.

In summary, one in ten adults in Europe, Canada and Israel likely have CKD. Of those, two out of three have not been diagnosed with CKD, and many are not treated using RAAS inhibitors. There is considerable public health potential in diagnosing CKD using widely available low-cost testing. Physicians should recognize that cardiorenal outcomes are the major causes of morbidity and mortality in patients with CKD and focus on disease modifying therapies that target these outcomes. From a research perspective, clinical trials examining patients with CKD can use these estimates to predict event rates for individual outcomes as well as composites for designing these trials. From a policy perspective, economic analyses aimed at determining the cost-effectiveness or utility of programs that target CKD can use our findings to determine the potential impact and cost savings from interventions.

## Contributors

J.S., J.B., A.B., M.G.V., P.B.M., A.K., T.T.G., M.B., K.I.B., M.T., L.J., M.M.S., G.VP., and N.T. participated in the conceptualization of the study. Data curation and statistical analyses were separately performed in each country with oversight form each author in Belgium, Canada, Germany, Israel, The Netherlands, Norway, Portugal, Spain, Sweden, Switzerland, and the United Kingdom. J.S., J.B. and M.T. verified the data from each country. M.T. performed the data curation and formal statistical analyses of the data from all countries in discussion with all authors. J.S., J.B., A.B., M.G.V., P.B.M., A.K., T.T.G., M.B., K.I.B., M.T., L.J., M.M.S., G.VP., and N.T. participated in data interpretation and in writing the manuscript. J.S., N.V. and J.B. drafted the original manuscript with further review and editing from all authors.

## Data sharing statement

Data sources utilised in this project are subject to ethical and privacy restrictions in each participating country. Therefore, the data that support the findings of this study are not available on request.

## Declaration of interests

J.S. reports stock ownership in Anagram kommunikation AB and Symptoms Europe AB. J.B. holds a full-time position at AstraZeneca as an epidemiologist. A.B. reports no competing interests. M.G.V. has received personal fees from Amgen, Vifor, FMC, Otsuka, Medice, AstraZeneca. Grants received from Amgen, FMC, Dutch Kidney Foundation, European communion, Health Holland (Ministery of Economic affairs). Non-financial support received from FMC, Calcison, NedMag. P.B.M. reports lecture fees and travel to meetings support from Vifor, Astrazeneca, Pharmacomsos, Napp, Astellas, lecture fees from Novartis, Astellas and grants from Boehringer Ingelheim outside the submitted work. A.K. has received research grants and speaking honoraria from Astrazeneca, Novonordisk and Boehringer Ingelheim. T. T. G. declares speaker and consulting fees from AstraZeneca, BIAL, Daiichi-Sankyo, MSD, Medinfar and Novartis. TTG holds shares in MTG. M.B. has received honoraria from Astra Zeneca, Janssen, Lilly, Boehringer Ingelheim, Sanofi, Amgen and Novo Nordisk. K.I.B. has received grants to his institution from AstraZeneca for this study and for lectures and consulting from Novo Nordisk, Sanofi, Lilly, Boehringer Ingelheim and Merck Sharp & Dohme. M.T. holds a full-time position by an independent statistical consultant company, Statisticon AB, Uppsala, Sweden, of which AstraZeneca Nordic is a client. L.J. reports no competing interests. M.S. declares a speaker fee from AstraZeneca. G.V.P reports no competing interests. N.T. reports grants and personal fees from AstraZeneca, grants and personal fees from Janssen, grants and personal fees from BI-Lilly, grants and personal fees from Otsuka, grants, personal fees and other from Tricida, personal fees and other from Pulsedata, personal fees and other from Mesentech, personal fees and other from Renibus, other from ClinPredict, outside the submitted work; In addition, N.T. has a patent for A microfluidic device for point of care detection of urine albumin pending.

## Appendix Supplementary materials

Image, application 1
