# Supplementary material for: Prevalence, outcomes, and cost of chronic kidney disease in a contemporary population of 2·4 million patients from 11 countries: The CaReMe CKD study
Source: Lancet Reg Health Eur. 2022 Jun 30;20:100438. doi: 10.1016/j.lanepe.2022.100438 (PMC9459126; doi:10.1016/j.lanepe.2022.100438)
Supplement: Supplementary file 1 [file mmc1.docx]

**SUPPLEMENTARY MATERIAL**

**Contents**

[Investigator and Study Group 2](#_Toc101091080)

[CaReMe CKD Investigators (by country in alphabetical order) 2](#_Toc101091081)

[Contributing non-authors 2](#_Toc101091082)

[Data Sources 3](#_Toc101091083)

[*Belgium* 3](#_Toc101091084)

[*Canada* 3](#_Toc101091085)

[*Germany* 3](#_Toc101091086)

[*Israel* 4](#_Toc101091087)

[*The Netherlands* 4](#_Toc101091088)

[*Norway* 5](#_Toc101091089)

[*Portugal* 5](#_Toc101091090)

[*Spain* 5](#_Toc101091091)

[*Sweden* 5](#_Toc101091092)

[*Switzerland* 6](#_Toc101091093)

[*United Kingdom* 6](#_Toc101091094)

[Table S1: Samples of 2,275,062 identified by CKD diagnosis or one pathologic eGFR or UACR 7](#_Toc101091095)

[Table S2: CKD definitions 8](#_Toc101091096)

[Table S3: Index dates across countries for the three cohorts 9](#_Toc101091097)

[Table S4: Comorbidity definitions 10](#_Toc101091098)

[Table S5: Clinical outcomes 11](#_Toc101091099)

[Table S6. Prevalence - extended 12](#_Toc101091100)

[Table S7. Hospital health care costs (US$) per patient at index and cumulatively over 5-years 13](#_Toc101091101)

[Table S8. Prevalence of chronic kidney disease across five countries with data for both *Measured* and *Diagnosed CKD* 14](#_Toc101091102)

[Table S9. Baseline characteristics of patients with *Measured CKD* across five countries with data for both *Measured* and *Diagnosed CKD* 15](#_Toc101091103)

[Table S10. Baseline characteristics of patients with *Diagnosed CKD* across five countries with data for both *Measured* and *Diagnosed CKD* 16](#_Toc101091104)

# Investigator and Study Group

## CaReMe CKD Investigators (by country in alphabetical order)

From Belgium, Professor Gijs VanPottelbergh MD PhD, Ine Van den Wyngaert MD, and Pavlos Mamouris PhD, Department of Public Health and Primary Care, KUleuven, Leuven.

From Canada, Professor Navdeep Tangri MD PhD, Department of Medicine and Community Health Sciences, University of Manitoba, Winnipeg; Manish M Sood MD MSc, Ottawa Hospital Research Institute, University of Ottawa, Ottawa, Ontario; and Roshanak Mahdavi Msc, ICES, Ottawa Hospital Research Institute, Ottawa, Ontario.

From Germany, Professor Andreas Bollmann MD PhD, and Sebastian König PhD, Heart Center Leipzig at University of Leipzig and Leipzig Heart Institute, Leipzig; and Johannes Leiner PhD, Vincent Pellissier PhD, and Anne Nitsche PhD, Leipzig Heart Institute, Leipzig.

From Israel, Professor Avraham Karasik MD PhD, and Cheli Melzer Cohen MD PhD, Maccabi Institute for Research and Innovation, Maccabi Healthcare Services, Tel Aviv.

From The Netherlands, Professor Marc G Vervloet MD PhD, Amsterdam UMC, Department of Nephrology, Amsterdam, The Netherlands; Jetty A Overbeek PhD, and Hilda JI de Jong PhD, PHARMO Institute for Drug Outcomes Research, Utrecht.

From Norway, Professor Kåre I Birkeland MD PhD, Department of transplantation medicine, Oslo University Hospital and University of Oslo, Oslo, Oslo University Hospital and University of Oslo, Oslo; and Johan Bodegård MD PhD, AstraZeneca Nordic, Oslo.

From Portugal, Professor Tiago Taveira-Gomes MD PhD, Department of Community Medicine, Information and Decision in Health, Faculty of Medicine, University of Porto, Portugal, Faculty of Health Sciences, Fernando Pessoa University, University Institute of Health Sciences, Advanced Polytechnic and University Cooperative, CRL, Magalhães & Taveira-Gomes Sociedade Médica Lda.; and Professor Carla Santos Araújo, Nephrology Department, Hospital Pedro Hispano, ULSM, and Cardiovascular Research and Development Center, Faculty of Medicina of the University of Porto.

From Spain, Professor Manuel Botana MD, University Hospital Lucus Augusti, Lugo; Roberto Alcazar MD, University Hospital Infanta Leonor, Madrid; Antonio Hormigo MD, Primary Care Center Puerta Blanca, Malaga; and Nicolás Manito MD, University Hospital Bellvitge, Hospitalet de Llobregat, Barcelona.

From Sweden, Professor Johan Sundström MD PhD, Department of Medical Sciences, Uppsala University, Uppsala, and The George Institute for Global Health, University of New South Wales, Sydney, Australia; Thomas Cars PhD, Sence Research AB, Uppsala; and Marcus Thuresson PhD, Statisticon AB, Uppsala.

From Switzerland, Levy Jäger MD, Institute of Primary Care, University of Zurich and University Hospital Zurich, Zurich; and Professor Thomas Rosemann MD PhD, Institute of Primary Care, University of Zurich and University Hospital Zurich, Zurich.

From United Kingdom, Professor Patrick B Mark, Institute of Cardiovascular and Medical Sciences, University of Glasgow, Glasgow; and Ruiqi Zhang PhD, Medical and Scientific Affairs, BioPharmaceuticals Medical, AstraZeneca, Cambridge.

## Contributing non-authors

From Belgium, Isabelle Fovel, and Laurens Minerva, AstraZeneca, Brussels.

From Canada, Sheena Kayaniyil PhD, and Navid Shobeiri PhD, AstraZeneca, Ontario.

From Germany, Marija Halbach and Antje Arnold, AstraZeneca, Hamburg.

From Israel, Maya Greenbloom, AstraZeneca, Tel Aviv.

From The Netherlands, Professor Ron MC Herings MD PhD, Louise Lemmens PhD, and Fernie JA Penning-van Beest PhD, PHARMO Institute for Drug Outcomes Research, Utrecht; Martine Oudenhuijzen and Wendy Beekman-Hendriks PhD, AstraZeneca Netherlands, Den Haag.

From Portugal, João Couceiro, Hugo Martinho, and Filipa Bernardo, AstraZeneca, Lisbon.

From Spain, Carlos Escobar Cervantes MD, University Hospital La Paz, Madrid; and Beatriz Palacios PhD and Unai Aranda MSc, AstraZeneca, Madrid.

From Sweden, Susanna Jerström and Helena Goike PhD, AstraZeneca Nordic, Södertälje.

From Switzerland, Felix Hugentobler PhD, AstraZeneca, Baar.

From United Kingdom, Jil Billy Mamza PhD, AstraZeneca United Kingdom, Cambridge.

# Data Sources

The study consists of data from nationwide cohort studies including heart failure (CKD) patients from Belgium, Canada, Germany, Israel, Netherlands, Norway, Portugal, Spain, Sweden, Switzerland and United Kingdom to obtain a study population of appropriate coverage. Each country is represented by a national scientific committee. The initiative is sponsored by AstraZeneca. A description of the respective databases is provided below.

## *Belgium*

Data were obtained from the INTEGO, a Belgian General Practice-based morbidity registration network at the Department of General Practice of the University Hospital of Leuven. INTEGO is a large database as the result of continuous recording in a network of general practices since 1994 (626.228 unique patients (1994-2021), 15.210.278 diagnoses, 117.317.712 laboratory tests, 26.605.144 medication prescriptions and 700,000 vaccination data). This is the only operational computerized morbidity registration network in Belgium based on 104 general practices data. INTEGO procedures were approved by the ethical review board of the Medical School of KU Leuven (N° ML 1723) and by the Belgian Privacy Commission (no SCSZG/13/079).

##

## *Canada*

We conducted a retrospective cohort analysis using administrative health databases housed at the Repository at the Manitoba Centre for Health Policy (MCHP) at the University of Manitoba. The Repository holds population-wide de-identified health information for Manitoba residents. All databases are de-identified but contain a scrambled personal health identification number (PHIN) that allows linking unique individuals across databases. Demographics and vital status information were obtained from the Manitoba Health Insurance Registry. Medication information was obtained from the Drug Program Information Network (DPIN) database. Diagnostic and procedural information from all hospitalizations was determined using the Hospital Discharge Abstracts (CIHI-DAD). Laboratory data was obtained from the Diagnostic Services of Manitoba database which captures laboratory measures from hospital and community laboratories in Manitoba.

Similarly, ICES in Ontario holds comprehensive longitudinal data of every patient in Ontario, Canada since 1982. A research group from ICES conducted the same analysis using datasets that were linked using unique encoded identifiers in Ontario, Canada. We ascertained patient characteristics, medication data, and outcome data from linked databases using unique encoded identifiers and analyzed at ICES. Demographics and vital status information were obtained from the Ontario Registered Persons Database. Medication information was obtained from the Ontario Drug Benefit Claims (ODB) database. This database contains highly accurate records of all outpatient prescriptions dispensed to patients aged 65 or older, with an error rate of less than 1%. Diagnostic and procedural information from all hospitalizations was determined using the Canadian Institute for Health Information Discharge Abstract Database (CIHI-DAD). Diagnostic information from emergency room visits was determined using the National Ambulatory Care Reporting System (NACRS). Information was also obtained from the Ontario Health Insurance Plan (OHIP) database, which contains all health claims for inpatient and outpatient physician services. Laboratory information is contained in the Ontario Laboratory Information System (OLIS) which captures 95% of laboratory tests for patients in Ontario.

This study was approved by the University of Manitoba Health Research Ethics Board (ethics file number HS223414 (H2019:454)). The results and conclusions are those of the authors and no official endorsement by Manitoba Health was intended or should be inferred. Data used in this study are from the Population Health Research Data Repository housed at the Manitoba Centre for Health Policy, University of Manitoba and were derived from data provided by Manitoba Health, Seniors, and Active Living, Vital Statistics, and Shared Health Diagnostic Services.

This study was also supported by ICES, which is funded by an annual grant from the Ontario Ministry of Health and Long-term Care (MOHLTC). The research was conducted by members of the ICES Kidney, Dialysis and Transplantation team at the ICES Western and Ottawa sites. Core funding for ICES Western is provided by the Academic Medical Organization of Southwestern Ontario (AMOSO), the Schulich School of Medicine and Dentistry (SSMD), Western University, and the Lawson Health Research Institute (LHRI). Core funding for ICES Ottawa is supported by a grant from the Canadian Institutes of Health Research (CIHR). The analyses, conclusions, opinions and statements expressed herein are solely those of the authors and do not reflect those of the funding or data sources; no endorsement is intended or should be inferred. Parts of this material are based on data and/or information compiled and provided by the Canadian Institute for Health Information (CIHI). However, the analyses, conclusions, opinions and statements expressed in the material are those of the author(s), and not necessarily those of CIHI. We thank Service Ontario for use of Office of the Registrar General (ORG) information on deaths. The views expressed therein are ICES’ and do not necessarily reflect those of ORG or Ministry of Government Services. We thank IQVIA Solutions Canada Inc. for use of their Drug Information Database.

## *Germany*

The “HELIOS-CKD” study database includes CKD patient information from administrative data covering 86 Helios hospitals. Administrative data provides information on basic characteristics (age, gender), the encoded main and secondary diagnoses at hospital discharge, type of hospital admission and type of hospital discharge. EMR data contains additional information (including used medication and laboratory results) for the sub-cohort of patients from the Heart Center Leipzig. The data are arranged on a case-by-case basis and can only be assigned to the specific patient within one unique hospital. There are no cross-links between hospitals with regard to cases of individual patients.

## *Israel*

Data from the Maccabi Healthcare Services (MHS) will be used for this study. MHS is the second largest primary healthcare insurer and provider in Israel. This health maintenance organization (HMO) serves 25% of the total population in Israel, with approximately 2 million members. Since 1999, information on member–MHS interactions have been recorded in a large central computerized database. The database includes information on hospitalizations, emergency department visits, physician visits, outpatient specialist visits, home healthcare visits, purchases of medications and other aids, laboratory tests, imaging, and paramedical services such as nursing care, physiotherapy, social workers, and dietary consultations. The database includes several automatically formulated registries, including a diabetes, CKD and cardiovascular diseases registries. These registries are updated daily and automatically utilizing strict algorithm. Israel is well-represented by this database, though Maccabi patients are slightly younger, less heterogeneous, and have a slightly better higher income compared to the country as a whole.(1)

All demographic, administrative and clinical data were collected from MHS’ computerized databases. The databases include, beyond demographic and administrative data (age, sex, MHS enrollment date, date of birth, smoking status) comorbidities gathered in MHS chronic disease registries (diabetes mellitus, hypertension, chronic kidney disease (CKD), chronic obstructive pulmonary disease (COPD), and specific CVD registry) patient’s socioeconomic status (SES) and healthcare utilization data. Specific diagnoses are also available to include: cerebrovascular accident (CVA), transient ischemic attack (TIA), myocardial infarction (MI), peripheral vascular disease (PVD) and atrial fibrillation (AF).

##

## *The Netherlands*

The PHARMO Database Network is a population-based network of electronic healthcare databases combining data from different primary and secondary healthcare settings in the Netherlands. These different data sources, including data from general practices, in- and out-patient pharmacies, clinical laboratories, hospitals, the cancer registry, pathology registry and perinatal registry, are linked on a patient level through validated algorithms. To ensure the privacy of the data in the PHARMO Database Network, the collection, processing, linkage and anonymisation of the data is performed by STIZON. STIZON is an independent, ISO/IEC 27001 certified foundation, which acts as a Trusted Third Party (TTP) between the data sources and the PHARMO Institute. Detailed information on the methodology and the validation of the used record linkage method can be found elsewhere (2,3).

To address the objectives of the present study the following PHARMO databases were used:

- General Practitioner Database
- Hospital Database – *Hospital admissions*
- Out-patient Pharmacy Database

A detailed description of these databases is given below.

General Practitioner Database

The General Practitioner (GP) Database comprises data from electronic patient records registered by GPs. The records include information on diagnoses and symptoms, laboratory test results, referrals to specialists and healthcare product/drug prescriptions. The prescription records include information on type of product, prescription date, strength, dosage regimen, quantity and route of administration. Drug prescriptions are coded according to the WHO Anatomical Therapeutic Chemical (ATC) Classification System (4). Diagnoses and symptoms are coded according to the International Classification of Primary Care (ICPC) (5),which can be mapped to ICD codes, but can also be entered as free text. GP data cover a catchment area representing 3.2 million residents.

Hospital Database

The Hospital Database comprises of datasets containing data on hospital admissions, ambulatory consultations and high budget impact medication. For the present study the hospital admissions dataset was used. The Hospital Database is collected and maintained by the Dutch Hospital Data Foundation (6); and it comprises records from nearly all hospitals in the Netherlands. With permission from each hospital the data are linked for research purposes with the PHARMO Database Network via the TTP.

The hospital admissions dataset comprises hospital admissions for more than 24 hours and admissions for less than 24 hours for which a bed is required (i.e. in-patient records) from the Dutch Hospital Data Foundation. The records include information on hospital admission and discharge dates, discharge diagnoses and procedures. Ambulatory consultations are not included. Diagnoses are coded according to the WHO International Classification of Diseases (ICD) (7). Procedures are coded according to the Dutch Hospital Data Foundation registration system for procedures which links to the Dutch Healthcare Authority (NZa) declaration codes and the Dutch Classification of Procedures (8,9). The Dutch Hospital Data Foundation collects hospitalization records from nearly all hospitals in the Netherlands. With permission from each hospital the data are linked for research purposes with the PHARMO Database Network via a trusted third party. Currently, PHARMO has access to data from 1998 onwards and of over 80% of the hospitals in the Netherlands.

Out-patient Pharmacy Database

The Out-patient Pharmacy Database comprises GP or specialist prescribed healthcare products dispensed by the out-patient pharmacy. The dispensing records include information on type of product, date, strength, dosage regimen, quantity, route of administration, prescriber specialty and costs. Drug dispensing is coded according to the WHO Anatomical Therapeutic Chemical (ATC) Classification System (4). Out-patient pharmacy data cover a catchment area representing 4.2 million residents.

## *Norway*

The study database includes patient information from three linked national Norwegian registries with full coverage of the Norwegian population: the Norwegian Prescription Database (July 2004 to April 2020) covering all filled drug prescriptions using ATC codes; the Norwegian Cause of Death Registry (1958 to April 2020) (10); and the Norwegian Patient Register covering all open patient clinic visit diagnoses and all hospital discharge diagnoses for the years 2008 to 2020. Diagnoses are recorded according to the ICD-system. Data linkage was performed by the Norwegian Institute of Public Health.

The study was approved by the Regional Ethics Committee, Helse Sør-Øst (reference numbers 2015/1337/REK sør-øst A and 11744) and was authorized by the Norwegian Data Inspectorate (Datatilsynet). The linked database was separately managed by Statisticon AB (Uppsala, Sweden).

## *Portugal*

The Unidade de Saude Local de Matosinhos EPE (USLM-EPE), is an integrated public medical care centre comprising both primary, secondary and tertiary healthcare. It fully serves the population of Matosinhos region, a urban area, that amounts to approximately 175.000 patients. Data was collected and anonymised by the hospital IT department from multiple healthcare systems used to provide everyday care both in hospital and primary care.

The study was approved by the Ethics Committee and the Data Protection Officer of USLM-EPE. This was a secondary data study and data were fully anonymized and dissociated from patients. Therefore, according to Portuguese regulation, there was no need for collecting informed consent from the patients.

## *Spain*

Observational cohort study, comprising cross-sectional and longitudinal retrospective analyses using secondary data captured in electronic health records from seven Spanish regions. Data sources were provided by BIG-PAC®. BIG-PAC is an electronic database that integrates information from primary and specialist care medical records. This database has been validated as an information source for studies of epidemiology, therapeutic adaptation and health/non-healthcare resource use. It has been demonstrated its representativeness of the Spanish population (11).

This study was approved by the Investigation Ethics Committee of Consorci Sanitari from Terrassa on 16th December 2019. This was a secondary data study and data were fully anonymized and dissociated from patients. Therefore, according to Spanish regulation, there was no need for providing informed consent.

## *Sweden*

The CELOSIA CKD study database includes patient information from three linked national Swedish registries with virtually complete coverage of the Swedish population: the Prescribed Drug Register (2005 to 2021) covering all filled drug prescriptions using the Anatomical Therapeutic Chemical (ATC) codes; the Cause of Death Registry (1961 to2021); and the National Patient Registry covering all open patient clinic visit diagnoses for 2001 to 2021 and all hospital discharge diagnoses for the years 1987–2021 (12). Diagnoses are recorded according to the ICD-system and has been shown to be of high validity (13). All three national registers are held by the Swedish National Board of Health and Welfare (NBHW). Regional data on laboratory and clinical measurements from Electronic Health Records (EHRs) in Region Stockholm (2.4 million inhabitants; 24% of the Swedish population) and Region Skåne (1.34 million inhabitants;13% of the Swedish population) are also included. The data linkage of the study database was performed by NBHW by using unique personal identification numbers (14).

The CELOSIA CKD study population comprises:

1. All patients in Sweden with at least one diagnosis of CKD, kidney disease or diabetes mellitus recorded in the National Patient Register during the time period 01 January 2000 to 01 January 2019 or
2. All patients in Region Stockholm and Region Skåne with at least one of the following laboratory test results recorded in EHRs within the public healthcare during the time period of 2010 (2013 for Region Skåne) to 01 January 2019:

1. BNP (> 100 ng/l)
2. NT-proBNP (>300 ng/l)
3. eGFR (< 60 ml/min)
4. HbA1c (> 48 mmol/L)
5. UACR >3 mg/mmol

For the study population, the CELOSIA CKD dataset covers information on age, sex, diagnoses (ICD10), clinical procedure codes and drug utilization for all patients in Sweden as well as clinical measurements (such as height, weight, BMI, blood pressure, smoking habits) and results from laboratory tests for patients in Region Stockholm and Region Skåne.

The study was approved by the Ethical Review Authority (reference number 2020-03850). The CELOSIA dataset was separately managed by Sence Research AB (Uppsala, Sweden).

## *Switzerland*

Data for this study originates from the Family medicine International classification of primary care Research using Electronic medical records (FIRE) project hosted and managed by the Institute of Primary Care of the University of Zurich. Since start of the project in 2009, the FIRE database had collected routine clinical data (drug prescriptions as ATC codes, reasons for encounters as ICPC-2 codes, laboratory test results and vital parameters) from electronic medical records of 575 general practitioners in German-speaking Switzerland as of 01 January 2020. The local ethics committee of the Canton of Zurich waived approval for studies based on the FIRE database, (reference number BASEC-Nr. Req-2017-00797), as they lie outside the scope of the Federal Act on Research involving Human Beings.

## *United Kingdom*

Clinical Practice Research Datalink (CPRD) Aurum database contains data sourced from GP practices using EMIS® GP software and holds anonymized longitudinal primary care patient records collected from over 873 general practices across UK, covering >20 million patients (10% of English practices), of whom 7 million (13% of the population of UK) were alive and currently contributing to the database as of September 2018. It includes diagnoses, issued drug prescriptions, clinical measures taken within the general practice, lab tests and referrals to specialist care, and have been linked to national secondary care databases (e.g., Hospital Episode Statistics, HES with detailed hospitalisation information on hospital admissions episodes in UK) as well as deprivation and death registration (Office for National Statistics, ONS) data. CPRD Aurum is updated monthly with complete reliable data spanning from 1 January 1995. As of April 2019, there was a median follow-up of 4.8 years (IQR: 1.9–12.1) for all patients and 9.2 years (IQR: 3.4–20.6) for current patients. Hospitalisation information and specialist care notes are generally recorded by the general practitioner into the primary care patient records. All the patient records utilised in this study had linkage to HES Admitted Patient Care data and death registration data from the ONS data. The ONS mortality data was used to identify the mortality outcomes. CPRD Aurum includes approximately 12% of the UK population (currently contributing patients only), and patients are broadly representative of the UK general population (Dedman et al., 2019). Herrett *et al* and Wolf *et al* assessed the representativeness of CPRD database by comparing the age and sex distribution within CPRD to the UK Census in 2011 (15,16). They found that CPRD was broadly representative of the UK population with respect to age and sex. This generalizability of CPRD to the UK population has resulted in CPRD being used in over 1000 publications (15,16).

This overall study protocol was approved by the Independent Scientific Advisory Committee (ISAC) of CPRD; protocol reference number: 20_139. This study is based in part on data from the Clinical Practice Research Datalink (CPRD) obtained under license from the UK Medicines and Healthcare Products Regulatory Agency. The data is provided by patients and collected by the National Health Service as part of patient care and support. This CPRD study also used data from the Office for National Statistics and Hospital Episode Statistics. Copyright © (2019), reused with the permission of The Health & Social Care Information Centre. All rights reserved. The interpretation and conclusions contained in this study are those of the authors alone.

# Table S1: Samples of 2,275,062 identified by CKD diagnosis or one pathologic eGFR or UACR

|  | Belgium | Canada | Germany | Israel | The Netherlands | Norway | Portugal | Spain | Sweden | Switzerland | UK |
| --- | --- | --- | --- | --- | --- | --- | --- | --- | --- | --- | --- |
| Number of patients, n | 25,156 | 1,235,791 | 161,407 | 111,627 | 91,023 | 101,992 | 11,802 | 56,435 | 204,963 | 9,118 | 391,618 |
| Index year | 2020 | 2018 | 2019 | 2021 | 2019 | 2020 | 2019 | 2018 | 2019 | 2020 | 2019 |
| Age, years (SD) | 71 (16) | 69 (16) | 77 (11) | 70 (14) | 73 (12) | 71 (16) | 72 (14) | 76 (14) | 73 (16) | 77 (12) | 75 (14) |
| Females, n (%) | 15,150 (60) | 664,457 (54) | 81,193 (50) | 52,403 (47) | 51,479 (57) | 42,033 (41) | 6,184 (52) | 26,957 (48) | 104,318 (51) | 5,110 (56) | 225,933 (58) |
| CKD diagnosis, n (%) | 9,293 (37) | 421,795 (34) | 161,407 (100) | 27,868 (25) | n/a | 101,992 (100) | 2,377 (20) | 56,435 (100) | 68,639 (33) | n/a | 391,618 (100) |
| **Comorbidities** |  |  |  |  |  |  |  |  |  |  |  |
| Heart failure, n (%) | 1,730 (7) | 227,615 (18) | 63,683 (39) | 9,673 (9) | 10,556 (12) | 22,645 (22) | 1,575 (13) | 11,610 (21) | 36,245 (18) | n/a | 65,748 (17) |
| Coronary ischemic disease, n (%) | n/a | 302,186 (24) | 21,695 (13) | 13,261 (12) | 21,628 (24) | 29,917 (29) | 1,199 (10) | 10,519 (19) | 46,715 (23) | n/a | 123,088 (31) |
| Stroke, n (%) | 1,357 (5) | 89,248 (7) | 13,328 (8) | 5,149 (5) | 15,126 (17) | 3,085 (3) | 1,529 (13) | 5,967 (11) | 32,579 (16) | n/a | 62,720 (16) |
| Atrial fibrillation/flutter, n (%) | 3,138 (12) | 126,438 (10) | 51,717 (32) | 11,673 (10) | 14,263 (16) | 26,744 (26) | 1,433 (12) | 8,921 (16) | 46,215 (23) | n/a | 79,006 (20) |
| Peripheral artery disease, n (%) | 1,298 (5) | 23,219 (2) | 14,817 (9) | 5,470 (5) | 19,669 (22) | 9,921 (10) | 380 (3) | 2,700 (5) | 10,696 (5) | n/a | 26,794 (7) |
| Diabetes, n (%) | 5,099 (20) | 489,230 (40) | 61,029 (38) | 49,313 (44) | 7,136 (8) | 25,938 (25) | 5,461 (46) | 27,394 (49) | 60,180 (29) | 2,145 (24) | 112,196 (29) |
| Cancer, n (%) | 6,708 (27) | 383,548 (31) | 18,694 (12) | 26,733 (24) | 14,003 (15) | 29,405 (29) | 1,281 (11) | 9,026 (16) | 40,709 (20) | n/a | 59,760 (15) |
| **Laboratory measurements** |  |  |  |  |  |  |  |  |  |  |  |
| SBP, mmHg, mean (SD) | n/a | n/a | n/a | 133·5 (16·9) | 138·0 (17·0) | n/a | 137·3 (16·9) | 137·6 (20·2) | 137·0 (19·6) | 137·4 (20·0) | 132·6 (15·8) |
| Sodium, mmol/L, mean (SD) | n/a | 140·3 (3·2) | n/a | 139·3 (2·5) | n/a | n/a | 139·2 (3·1) | 138·5 (12·6) | n/a | 140·4 (3·2) | 139·9 (3·5) |
| Potassium, mmol/L, mean (SD) | 4·5 (0·6) | 4·4 (0·5) | n/a | 4·6 (0·7) | 4·3 (0·4) | n/a | 4·4 (0·5) | 4·0 (0·8) | 4·4 (0·6) | 4·3 (0·4) | 4·6 (0·5) |
| >5.5 mmol/L, n (%) | 1,029 (6) | 18,680 (2) | n/a | 4,710 (5) | 443 (1) | n/a | 224 (3) | 3,395 (6) | 6,373 (5) | 122 (2) | 11,042 (3) |
| Magnesium, mmol/L, mean (SD)) | n/a | 0·8 (0·1) | n/a | 0·8 (0·1) | n/a | n/a | 0·8 (0·1) | n/a | n/a | 0·8 (0·1) | 0·8 (0·5) |
| Calcium, mmol/L, mean (SD) | n/a | 2·3 (0·3) | n/a | 2·3 (0·6) | n/a | n/a | 2·3 (0·2) | 9·0 (0·9) | n/a | 2·4 (0·1) | 2·4 (6·5) |
| eGFR, mL/min/1.73 m^2^, mean (SD) | 65·9 (20·8) | 62·8 (NA) | n/a | 67·3 (25·3) | 65·0 (20·0) | n/a | 60·5 (23·3) | 49·8 (20·0) | 56·1 (19·9) | 51·0 (18·7) | 52·2 (15·8) |
| Creatinine, mg/dL, mean (SD) | n/a | 1·2 (0·8) | n/a | 1·2 (2·7) | 1·1 (0·4) | n/a | 1·2 (0·6) | 1·3 (0·6) | 1·2 (0·7) | 1·1 (0·3) | 1·3 (0·8) |
| S-Albumin, g/dL, mean (SD) | n/a | 4·0 (0·5) | n/a | 4·1 (0·4) | n/a | n/a | 4·0 (0·6) | n/a | n/a | 42·3 (4·0) | 4·0 (0·5) |
| uACR, mg/g, mean (SD) | 141·0 (427·2) | 21·3 (68·5) | n/a | 91·5 (101·4) | 61·9 (265·5) | n/a | 124·8 (446·4) | 390·8 (300·0) | 161·5 (525·0) | 61·2 (52·7) | 117·3 (470·1) |
| % of patients with measurement | 7·7 | 56·6 | n/a | 95·1 | 76.4 | n/a | 89·9 | 100·0 | 43·0 | 31·5 | 42·1 |
| HbA1c DCCT, %, mean (SD) | 6·4 (1·0) | 6·4 (1·4) | n/a | 6·3 (1·3) | 6·0 (1·6) | n/a | 6·7 (1·4) | 6·8 (1·2) | n/a | 6·0 (0·8) | 6·2 (1·2) |
| Hemoglobin, g/dL, mean (SD) | 13·6 (1·6) | 13·2 (1·8) | n/a | 13·4 (1·8) | n/a | n/a | 13·2 (1·8) | 13·4 (1·3) | 13·2 (1·7) | 13·3 (1·6) | 13·1 (1·7) |
| Hb 10-12, g/dL, n (%) | 2,771 (12) | n/a | n/a | 18,121 (16) | n/a | n/a | 2,152 (20) | 3,941 (7) | 35,394 (20) | 1,520 (18) | 63,259 (20) |
| Hb ≤10, g/dL | 460 (2) | n/a | n/a | 3,409 (3) | n/a | n/a | 492 (5) | 3,034 (5) | 6,868 (4) | 322 (4) | 11,500 (4) |
| Hematocrit, %, mean (SD) | 40·8 (20·8) | 39·7 (4·9) | n/a | 41·4 (5·1) | 41·0 (5·0) | n/a | n/a | 40·4 (10·9) | n/a | 39·4 (4·6) | 39·9 (4·8) |
| <40% | 8,710 (39) | n/a | n/a | 41,755 (38) | 13,228 (37) | n/a | n/a | 4,896 (9) | n/a | 4,469 (53) | 146,214 (49) |
| **CKD treatment, n (%)** | 9,816 (39) | 528,943 (43) | n/a | 74,382 (67) | 51,075 (56) | n/a | 8,267 (70) | n/a | 121,003 (59) | n/a | 183,483 (47) |
| RAAS inhibitor | 9,001 (36) | 514,803 (42) | n/a | 72,087 (65) | 49,464 (54) | 52,284 (51) | 8,206 (70) | 39,046 (69) | 116,021 (57) | 5,431 (60) | 179,043 (46) |
| MRA | 1,682 (7) | 32,549 (3) | n/a | 6,829 (6) | 4,701 (5) | 6,178 (6) | 771 (7) | 3,336 (6) | 15,937 (8) | 548 (6) | 14,549 (4) |
| SGLT-2i | 26 (0) | 33,664 (3) | n/a | 5,424 (5) | 591 (1) | 2,991 (3) | 579 (5) | 889 (2) | 3,794 (2) | 269 (3) | 2,947 (1) |
| Dialysis | n/a | 16,852 (1) | 6,368 (4) | 1,628 (1) | 568 (1) | 5,541 (5) | n/a | 925 (2) | 5,193 (3) | n/a | 4,011 (1) |

SD, Standard deviation. SBP, systolic blood pressure. All numbers in parenthesis are percentage if not stated otherwise. *Random effect estimates. CKD, chronic kidney disease. RAAS, renin angiotensin aldosterone system. MRA, mineralocorticoid receptor antagonist. SGLT-2i, sodium-glucose-cotransporter-2-inhibitors. DCCT, Diabetes Control and Complications Trial units. UK, United Kingdom.

# Table S2: CKD definitions

| Name | **Definition** |
| --- | --- |
| Possible CKD | One CKD diagnosis or one pathological UACR or eGFR value |
| Measured CKD | Two pathological UACR or eGFR values at least 90 days apart |
| Diagnosed CKD | At least one registered CKD diagnosis prior to index, with or without eGFR and/or UACR |
| Single-measure CKD | One pathological UACR or eGFR value |
| Time-limited CKD | CKD measured defined within the last two years |
| Persistent CKD | Two pathological UACR or eGFR values at least 90 days apart with no normal values in-between |

CKD, chronic kidney disease. eGFR, estimated glomerular filtration rate (ml/min/1.73 m2). UACR, Urine albumin-to-creatinine ration.

Pathological value defined as eGFR <60 ml/min/1.73 m2 or UACR ≥30 mg/g (≥3 mg/mmol).

# Table S3: Index dates across countries for the three cohorts

|  | **Cohort 1:**  Most contemporary baseline date | **Cohort 2:**  1-year event rate baseline date | **Cohort 3:**  5-year cost analysis baseline date |
| --- | --- | --- | --- |
| **Belgium** | 2020-01-01 |  |  |
| **Canada** | 2018-01-01 | 2017-01-01 | 2014-01-01 |
| **Germany** | 2019-01-01 | 2018-01-01 |  |
| **Israel** | 2021-01-01 | 2020-01-01 | 2016-01-01 |
| **The Netherlands** | 2019-01-01 | 2017-12-31 | 2014-12-31 |
| **Norway** | 2020-01-01 | 2019-01-01 | 2015-01-01 |
| **Portugal** | 2019-12-31 | 2018-12-31 | 2016-12-31 |
| **Spain** | 2018-01-01 | 2018-01-01 | 2015-01-01 |
| **Sweden** | 2019-01-01 | 2018-01-01 | 2015-01-01 |
| **Switzerland** | 2020-01-01 |  |  |
| **United Kingdom** | 2019-06-01 | 2018-06-01 | 2014-06-01 |

# Table S4: Comorbidity definitions

| Disease | ICD-8 | ICD-9 | ICD 10 | Surgical code/medication |
| --- | --- | --- | --- | --- |
| **CVRD (includes all codes below)** |  |  |  |  |
| Myocardial infarction | 410.9, 410.99 | 410 | I21-I22, I25.2, I25.6 |  |
| CABG |  | 414.02-07, V45.81-82 |  | Surgical by-pass codes |
| PCI with stent |  |  |  | Peripheral intervention codes |
| Unstable angina |  | 411 | I20.0 |  |
| Angina pectoris | 4193, 4139 | 413, 414.0 | I20.1, I20.8, I20.9, I25.1, I25.5 | Nitrates: C01DA |
| **Heart failure (total)** | 425.99, 427.09–427.19, 427.99, 428.99 | 428 | I50, I11.0, I13.0, I13.2 |  |
| Heart failure |  |  | I50 |  |
| Heart failure - hypertensive |  |  | I11.0, I13.0, I13.2 |  |
| **CKD (total)** | 581.00–582.09, 583 | 585, 583.81, 250D | N17-N19, I12.0-I12.9, I13.1, I13.2, N08.3, E10.2, E11.2, E12.2, E13.2, E14.2, Z49, Z99.2 | Dialysis and kidney transplantation codes |
| CKD - Acute |  |  | N17 |  |
| CKD - Chronic |  |  | N18 |  |
| CKD - Unspecified |  |  | N19 |  |
| CKD - Diabetic |  |  | E10.2, E11.2, E12.2, E13.2, E14.2, N08.3 |  |
| CKD - Hypertensive |  |  | I12.0-I12.9, I13.1, I13.2 |  |
| CKD - Dialysis |  |  | Z49, Z99.2 | Daily codes |
| Atrial fibrillation | 427.93, 427.94 | 427.3 | I48 |  |
| Stroke |  | 430-438, V125 | I60-I66, G45 |  |
| Hemorrhagic | 43000-43099, 43100, 43108–43190, 43198-43199 | 430-432 | I60-I62 |  |
| Ischemic | 43200–43299, 43309–43399, 43409-43499 | 433-434, 436 | I63 |  |
| Transitory ischemic attack | 43509-43599 | V12.5, 435 | G45 |  |
| Peripheral artery disease | 440.20–440.30 | 440/441/444 | I70.2, I73.9, I74.2-9 | Revascularization codes, upper/lower extremities |
| Dialysis |  |  | Z49, Z99.2 |  |
| Cancer | 140.0–204.4 | 140-239 | C00-C99 |  |

CVRD, cardiovascular renal disease. CABG, coronary artery by-pass grafting. CKD, chronic kidney disease. PCI, percutaneous coronary intervention.

# Table S5: Clinical outcomes

| Variable | Definition | Comment |
| --- | --- | --- |
| All-cause death | Death of any cause |  |
| Cardiovascular death | Death with any “I” diagnosis as underlying cause of death | Only in countries with cause of death registry |
| Renal death | Death with any “N” diagnosis as underlying cause of death |  |
| Myocardial infarction | I21, I22 |  |
| Stroke | I60-I63 |  |
| Heart failure | I50, I11.0, I13.0, I13.2 |  |
| Chronic kidney disease | N17-N19, I12.0-I2.9, I13.1, I13.2, N08.3, E10.2, E11.2, E12.2, E13.2, E14.2, Z49, Z99.2 + procedure codes |  |
| Peripheral artery disease | I70.2, I73.9, I74.2-9 |  |

# Table S6. Prevalence - extended

|  | Belgium | Canada | Israel | Portugal | Sweden | Pooled prevalence | Tau |
| --- | --- | --- | --- | --- | --- | --- | --- |
| **Prevalence** |  |  |  |  |  |  |  |
| Single-measure CKD | 10·3% | 8·5% | 8·6% | 10·9% | 6·9% | 9·0 (7·6‒10·4) | 1·59 |
| Measured CKD | 5·6% | 7·0% | 6·5% | 9·8% | 6·1% | 7·0 (5·6‒8·5) | 1·65 |
| Time-limited CKD | 3·2% | 4·4% | 4·3% | 9·8% | 4·6% | 5·3 (3·0‒7·5) | 2·58 |
| Persistent CKD | 2·9% | 4·5% | 5·6% | 9·8% | 5·4% | 5·6 (3·4‒7·8) | 2·54 |
| **Number of patients** |  |  |  |  |  |  |  |
| Single-measure CKD, n | 21,593 | 1,068,917 | 111,627 | 11,574 | 177,306 |  |  |
| Measured CKD, n | 11,744 | 883,310 | 84,229 | 10,455 | 156,230 |  |  |
| Time-limited CKD, n | 6,722 | 547,105 | 56,196 | 10,415 | 118,622 |  |  |
| Persistent CKD, n | 6,011 | 569,794 | 72,180 | 10,397 | 137,585 |  |  |
| Background population | 208,921 | 12,553,761 | 1,298,633 | 106,482 | 2,570,327 |  |  |

Random effects models were used to calculate pooled values, and the heterogeneity measure Ʈ (tau) corresponds to the estimated standard deviation of the underlying data. CKD, chronic kidney disease; eGFR, estimated glomerular filtration rate (ml/min/1.73m^2^). UACR, Urine albumin-to-creatinine ration; UK, United Kingdom.

# Table S7. Hospital health care costs (US$) per patient at index and cumulatively over 5-years

|  | **Year 1** | **Year 2** | **Year 3** | **Year 4** | **Year 5** |
| --- | --- | --- | --- | --- | --- |
| **Canada, n=1,235,849** |  |  |  |  |  |
| Chronic kidney disease | 1051 | 2057 | 3027 | 3959 | 4554 |
| Heart failure | 588 | 1126 | 1622 | 2096 | 2384 |
| Myocardial infarction | 217 | 412 | 590 | 743 | 827 |
| Stroke | 154 | 286 | 422 | 545 | 627 |
| Peripheral artery disease | 61 | 112 | 160 | 202 | 228 |
| **Portugal, n=11,802** |  |  |  |  |  |
| Chronic kidney disease | 328 | 693 | 1060 |  |  |
| Heart failure | 282 | 527 | 775 |  |  |
| Myocardial infarction | 83 | 144 | 215 |  |  |
| Stroke | 84 | 149 | 224 |  |  |
| Peripheral artery disease | 37 | 78 | 117 |  |  |
| **Spain, n=56,435** |  |  |  |  |  |
| Chronic kidney disease | 1084 | 2082 | 2985 | 3612 | 4441 |
| Heart failure | 1664 | 3139 | 4550 | 5775 | 6888 |
| Myocardial infarction | 82 | 149 | 210 | 270 | 342 |
| Stroke | 123 | 232 | 348 | 452 | 561 |
| Peripheral artery disease | 60 | 105 | 151 | 196 | 239 |
| **United Kingdom, n=391,618** |  |  |  |  |  |
| Chronic kidney disease | 1353 | 2844 | 4617 | 7297 | 10048 |
| Heart failure | 485 | 1022 | 1683 | 2731 | 3837 |
| Myocardial infarction | 417 | 852 | 1335 | 2073 | 2791 |
| Stroke | 151 | 293 | 461 | 724 | 1000 |
| Peripheral artery disease | 119 | 248 | 401 | 652 | 925 |

SD, Standard deviation

# Table S8. Prevalence of chronic kidney disease across five countries with data for both *Measured* and *Diagnosed CKD*

|  | **Canada** | **Israel** | **The Netherlands** | **Portugal** | **Sweden** | **Pooled prevalence** | **Tau** |
| --- | --- | --- | --- | --- | --- | --- | --- |
| **Prevalence** |  |  |  |  |  |  |  |
| Possible CKD | 9.8% | n/a | 8.9% | 11.1% | 8.3% | 9.5% (8.3‒10.7) | 1.22 |
| Measured CKD | 7.0% | 6.5% | n/a | 9.8% | 6.1% | 7.4% (5.7‒9.0) | 1.69 |
| *UACR CKD (Stage I-II)* | 2.4% | 2.8% | n/a | 3.5% | 1.4% | 2.5% (1.7‒3.4) | 0.86 |
| *eGFR CKD (Stage III-V)* | 4.7% | 3.7% | n/a | 6.3% | 4.6% | 4.8% (3.8‒5.9) | 1.07 |
| Diagnosed CKD | 3.4% | 2.1% | n/a | 1.8% | 3.7% | 2.8% (1.9‒3.7) | 0.92 |
| **Number of patients** |  |  |  |  |  |  |  |
| Possible CKD, n | 1,235,791 | n/a | 194,978 | 11,802 | 212,846 |  |  |
| Measured CKD, n | 883,310 | 84,229 | n/a | 10,455 | 156,230 |  |  |
| Diagnosed CKD, n | 421,795 | 27,868 | n/a | 1,932 | 95,575 |  |  |
| **Background population** | 12,553,761 | 1,298,633 | 2,187,962 | 106,482 | 2,570,327 |  |  |

# Table S9. Baseline characteristics of patients with *Measured CKD* across five countries with data for both *Measured* and *Diagnosed CKD*

|  | **Canada** | **Israel** | **The Netherlands** | **Portugal** | **Sweden** | **Pooled baseline value** | **Tau** |
| --- | --- | --- | --- | --- | --- | --- | --- |
| **Number of patients, n** | 883,310 | 84,229 | 49,413 | 10,455 | 156,230 | n/a | n/a |
| **Index year** | 2018 | 2021 | 2019 | 2019 | 2019 |  |  |
| **Age, years (SD)** | 72 (15) | 72 (13) | 76 (11) | 73 (14) | 75 (14) | 73.6 (71.9‒75.2) | 1.89 |
| **Females, n (%)** | 475,801 (54) | 37,700 (45) | 26,713 (54) | 5,471 (52) | 78,486 (50) | 51.0 (47.7‒54.4) | 3.84 |
| **CKD diagnosis, n (%)** | 251,305 (28) | 26,741 (32) | 24,007 (49) | 2,054 (20) | 56,398 (36) | 32.9 (23.6‒42.2) | 10.64 |
| **Comorbidities** |  |  |  |  |  |  |  |
| **Heart failure, n (%)** | 186,593 (21) | 8,682 (10) | 7,436 (15) | 1,403 (13) | 37,836 (24) | 16.8 (11.8‒21.8) | 5.71 |
| **Coronary ischemic disease, n (%)** | 247,748 (28) | 11,282 (13) | 13,741 (28) | 1,075 (10) | 42,537 (27) | 21.4 (13.7‒29.0) | 8.76 |
| **Stroke, n (%)** | 71,403 (8) | 4,424 (5) | 9,329 (19) | 1,340 (13) | 29,531 (19) | 12.8 (7.4‒18.2) | 6.19 |
| **Atrial fibrillation/flutter, n (%)** | 101,885 (12) | 10,128 (12) | 9,345 (19) | 1,272 (12) | 41,763 (27) | 16.3 (10.5‒22.1) | 6.59 |
| **Peripheral artery disease, n (%)** | 18,478 (2) | 4,621 (5) | 12,496 (25) | 344 (3) | 11,114 (7) | 8.7 (0.3‒17.0) | 9.50 |
| **Diabetes, n (%)** | 395,035 (45) | 41,554 (49) | 13,995 (28) | 4,885 (47) | 59,667 (38) | 41.5 (34.1‒48.8) | 8.42 |
| **Cancer, n (%)** | 293,605 (33) | 22,032 (26) | 8,052 (16) | 1,110 (11) | 38,850 (25) | 22.2 (14.5‒30.0) | 8.85 |
| **Laboratory measurements** |  |  |  |  |  |  |  |
| **Systolic blood pressure, mm Hg, mean (SD)** | n/a | 133.9 (16.7) | 138.0 (17.0) | 137.5 (17.0) | 137.2 (19.4) | 136.7 (134.8‒138.5) | 1.87 |
| **Sodium, mmol/L, mean (SD)** | 140.3 (3.2) | 139.3 (2.5) | n/a | 139.2 (3.1) | 139.9 (3.0) | 139.7 (139.2‒140.2) | 0.52 |
| **Potassium, mmol/L, mean (SD)** | 4.4 (0.5) | 4.7 (0.5) | 4.3 (0.4) | 4.4 (0.5) | 4.5 (0.6) | 4.5 (4.3‒4.6) | 0.15 |
| **>5.5 mmol/L, n (%)** | 15,879 (2) | 4,292 (6) | 358 (1) | 204 (3) | 5,744 (5) | 3.3 (1.5‒5.2) | 2.11 |
| **Magnesium, mmol/L, mean (SD))** | 0.8 (0.1) | 0.8 (0.1) | n/a | 0.8 (0.1) | 0.8 (0.2) | 0.8 (0.8‒0.8) | 0.01 |
| **Calcium, , mmol/L, mean (SD)** | 2.3 (0.3) | 2.3 (0.7) | n/a | 2.3 (0.2) | n/a | 2.3 (2.3‒2.3) | 0.02 |
| **eGFR, mL/min/1.73 m2, mean (SD)** | 58.9 (24.2) | 63.1 (24.7) | 56.0 (19.0) | 59.8 (23.7) | 53.7 (19.4) | 58.3 (55.1‒61.5) | 3.61 |
| **<15** | 16,880 (2) | 1,641 (2) | 291 (1) | 184 (2) | 2,237 (1) | 1.6 (1.1‒2.0) | 0.56 |
| **15-29** | 48,046 (6) | 3,704 (4) | 2,270 (5) | 716 (7) | 11,444 (8) | 5.8 (4.6‒7.0) | 1.36 |
| **30-44** | 163,402 (19) | 13,011 (15) | 9,101 (19) | 1,896 (18) | 31,400 (21) | 18.5 (16.8‒20.3) | 1.99 |
| **45-59** | 341,661 (40) | 30,104 (36) | 23,353 (49) | 3,916 (37) | 69,231 (46) | 41.6 (36.6‒46.6) | 5.70 |
| **60-89** | 166,153 (19) | 20,677 (25) | 9,360 (20) | 2,197 (21) | 27,417 (18) | 20.6 (18.4‒22.7) | 2.44 |
| **90+** | 121,686 (14) | 15,092 (18) | 3,211 (7) | 1,546 (15) | 8,582 (6) | 11.9 (7.2‒16.6) | 5.35 |
| **Creatinine, mg/dL, mean (SD)** | 1.3 (0.9) | 1.2 (3.0) | 1.2 (0.5) | 1.2 (0.6) | 1.2 (0.8) | 1.2 (1.2‒1.2) | 0.04 |
| **S-Albumin, g/dL, mean (SD)** | 4.0 (0.5) | 4.0 (0.4) | n/a | 4.0 (0.5) | n/a | 4.0 (4.0‒4.0) | 0.00 |
| **uACR, mg/g, mean (SD)** | 24.4 (73.3) | 97.6 (106.7) | 87.9 (322.2) | 128.8 (396.6) | 175.4 (546.3) | 102.8 (54.1‒151.5) | 55.54 |
| **% of patients with measurement** | 62.0 | 95.5 | 78.4 | 90.3 | 50.7 | 75.4 (58.8‒91.9) | 18.89 |
| **HbA1c DCCT, %, mean (SD)** | 6.5 (1.4) | 6.3 (1.3) | 6.6 (1.2) | 6.8 (1.4) | 6.5 (1.4) | 6.5 (6.4‒6.7) | 0.16 |
| **Hemoglobin, g/dL, mean (SD)** | 13.1 (1.8) | 13.3 (1.8) | 13.4 (1.6) | 13.2 (1.8) | 13.2 (1.7) | 13.2 (13.1‒13.3) | 0.13 |
| **Hb 10-12, g/dL, n (%)** | n/a | 15,187 (18) | 4,438 (16) | 1,933 (20) | 30,034 (21) | 18.6 (16.3‒20.9) | 2.32 |
| **Hb ≤10, g/dL** | n/a | 2,908 (3) | 805 (3) | 420 (4) | 5,627 (4) | 3.6 (3.0‒4.3) | 0.64 |
| **Hematocrit, %, mean (SD)** | 39.4 (4.9) | 41.1 (5.2) | 40.0 (5.0) | n/a | n/a | 40.2 (39.2‒41.1) | 0.84 |
| **<40%** | n/a | 33,340 (40) | 8,580 (42) | n/a | n/a | 40.7 (39.1‒42.4) | 1.17 |
| **CKD treatment n (%)** | 432,124 (49) | 60,678 (72) | 30,442 (62) | 7,356 (70) | 98,666 (63) | 63.2 (55.2‒71.2) | 9.16 |
| **RAS inhibitor** | 420,463 (48) | 58,735 (70) | 29,321 (59) | 7,302 (70) | 94,524 (61) | 61.4 (53.4‒69.4) | 9.17 |
| **MRA** | 27,332 (3) | 5,931 (7) | 3,297 (7) | 685 (7) | 13,628 (9) | 6.4 (4.6‒8.2) | 2.05 |
| **SGLT-2i** | 28,150 (3) | 4,732 (6) | 287 (1) | 521 (5) | 3,031 (2) | 3.3 (1.4‒5.1) | 2.09 |
| **Dialysis** | 14,837 (2) | 1,538 (2) | 505 (1) | n/a | 4,394 (3) | 1.8 (1.1‒2.6) | 0.74 |

# Table S10. Baseline characteristics of patients with *Diagnosed CKD* across five countries with data for both *Measured* and *Diagnosed CKD*

|  | **Canada** | **Israel** | **The Netherlands** | **Portugal** | **Sweden** | **Pooled baseline value** | **Tau** |
| --- | --- | --- | --- | --- | --- | --- | --- |
| **Number of patients, n** | 421,795 | 27,868 | 33,723 | 1,932 | 95,575 | n/a | n/a |
| **Index year** | 2018 | 2021 | 2019 | 2019 | 2019 |  |  |
| **Age, years (SD)** | 68 (17) | 75 (12) | 75 (12) | 78 (11) | 68 (19) | 72.6 (68.7‒76.5) | 4.48 |
| **Females, n (%)** | 206,645 (49) | 10,427 (37) | 18,672 (55) | 965 (50) | 46,052 (48) | 48.0 (42.2‒53.7) | 6.55 |
| **CKD diagnosis, n (%)** | 421,795 (100) | 27,868 (100) | 33,723 (100) | 1,932 (100) | 95,575 (100) | 100.0 (100.0‒100.0) | 0.00 |
| **Comorbidities** |  |  |  |  |  |  |  |
| **Heart failure, n (%)** | 113,464 (27) | 4,963 (18) | 5,365 (16) | 750 (39) | 21,284 (22) | 24.3 (16.3‒32.3) | 9.07 |
| **Coronary ischemic disease, n (%)** | 126,325 (30) | 5,032 (18) | 9,222 (27) | 440 (23) | 22,339 (23) | 24.3 (20.3‒28.3) | 4.57 |
| **Stroke, n (%)** | 40,780 (10) | 2,014 (7) | 6,415 (19) | 461 (24) | 15,750 (16) | 15.2 (9.3‒21.2) | 6.77 |
| **Atrial fibrillation/flutter, n (%)** | 62,629 (15) | 4,529 (16) | 6,410 (19) | 535 (28) | 20,915 (22) | 19.9 (15.5‒24.3) | 5.01 |
| **Peripheral artery disease, n (%)** | 13,027 (3) | 2,112 (8) | 8,701 (26) | 183 (9) | 6,602 (7) | 10.6 (2.8‒18.3) | 8.83 |
| **Diabetes, n (%)** | 162,212 (38) | 14,673 (53) | 8,280 (25) | 1,093 (57) | 33,507 (35) | 41.4 (29.9‒52.9) | 13.11 |
| **Cancer, n (%)** | 145,254 (34) | 8,260 (30) | 5,610 (17) | 389 (20) | 20,099 (21) | 24.4 (17.9‒30.9) | 7.38 |
| **Laboratory measurements** |  |  |  |  |  |  |  |
| **Systolic blood pressure, mm Hg, mean (SD)** | n/a | 133.4 (17.3) | 137.0 (17.0) | 136.9 (18.0) | 135.1 (20.3) | 135.6 (133.9‒137.2) | 1.69 |
| **Sodium, mmol/L, mean (SD)** | 140.2 (3.4) | 139.3 (2.6) | n/a | 139.3 (3.4) | 139.9 (3.0) | 139.7 (139.3‒140.1) | 0.43 |
| **Potassium, mmol/L, mean (SD)** | 4.4 (0.5) | 4.8 (0.5) | 4.3 (0.4) | 4.5 (0.6) | 4.5 (0.7) | 4.5 (4.3‒4.7) | 0.19 |
| **>5.5 mmol/L, n (%)** | 8,927 (2) | 2,888 (11) | 301 (1) | 91 (5) | 4,692 (8) | 5.6 (1.9‒9.2) | 4.17 |
| **Magnesium, mmol/L, mean (SD))** | 0.8 (0.1) | 0.8 (0.1) | n/a | 0.8 (0.1) | 0.8 (0.2) | 0.8 (0.8‒0.8) | 0.01 |
| **Calcium, , mmol/L, mean (SD)** | 2.2 (0.3) | 2.3 (1.2) | n/a | 2.3 (0.2) | n/a | 2.3 (2.2‒2.3) | 0.03 |
| **eGFR, mL/min/1.73 m2, mean (SD)** | 57.6 (28.1) | 45.1 (18.9) | 51.0 (16.0) | 42.2 (19.5) | 55.1 (24.2) | 50.2 (44.5‒55.9) | 6.48 |
| **Creatinine, mg/dL, mean (SD)** | 1.5 (1.3) | 1.8 (5.2) | 1.3 (0.6) | 1.7 (0.8) | 1.3 (1.0) | 1.5 (1.3‒1.7) | 0.22 |
| **S-Albumin, g/dL, mean (SD)** | 3.9 (0.6) | 4.0 (0.4) | n/a | 3.9 (0.6) | n/a | 3.9 (3.8‒4.0) | 0.07 |
| **uACR, mg/g, mean (SD)** | 36.3 (101.9) | 111.2 (122.1) | 87.2 (341.3) | 214.9 (635.7) | 264.1 (707.5) | 142.3 (59.7‒224.8) | 93.82 |
| **% of patients with measurement** | 58.3 | 95.2 | 70.1 | 90.7 | 40.2 | 70.9 (50.9‒90.9) | 22.83 |
| **HbA1c DCCT, %, mean (SD)** | 6.3 (1.4) | 6.3 (1.2) | 6.7 (1.1) | 6.8 (1.5) | 6.5 (1.4) | 6.5 (6.3‒6.7) | 0.21 |
| **Hemoglobin, g/dL, mean (SD)** | 12.7 (1.9) | 12.9 (1.9) | 13.3 (1.6) | 12.2 (1.8) | 13.0 (1.8) | 12.8 (12.5‒13.2) | 0.40 |
| **Hb 10-12, g/dL, n (%)** | n/a | 7,203 (26) | 3,355 (17) | 633 (34) | 18,478 (24) | 24.9 (18.1‒31.7) | 6.91 |
| **Hb ≤10, g/dL** | n/a | 1,769 (6) | 620 (3) | 208 (11) | 4,042 (5) | 6.4 (3.1‒9.6) | 3.30 |
| **Hematocrit, %, mean (SD)** | 38.6 (5.3) | 39.8 (5.5) | 40.0 (5.0) | n/a | n/a | 39.5 (38.6‒40.3) | 0.78 |
| **<40%** | n/a | 14,056 (51) | 6,290 (44) | n/a | n/a | 47.1 (40.2‒54.0) | 4.99 |
| **CKD treatment n (%)** | 175,178 (42) | 20,247 (73) | 20,113 (60) | 1,221 (63) | 51,472 (54) | 58.2 (48.0‒68.3) | 11.54 |
| **RAS inhibitor** | 168,724 (40) | 19,362 (69) | 19,326 (57) | 1,217 (63) | 49,220 (51) | 56.2 (46.4‒66.1) | 11.27 |
| **MRA** | 16,396 (4) | 2,779 (10) | 2,471 (7) | 221 (11) | 7,052 (7) | 8.0 (5.4‒10.5) | 2.85 |
| **SGLT-2i** | 8,265 (2) | 1,576 (6) | 102 (0) | 80 (4) | 1,414 (1) | 2.7 (0.8‒4.6) | 2.15 |
| **Dialysis** | 16,554 (4) | 1,500 (5) | 475 (1) | n/a | 5,073 (5) | 4.0 (2.2‒5.8) | 1.86 |

**Figure S1. Proportion of patients in the different chronic kidney disease (CKD) stages per across five countries with data for both *Measured* and *Diagnosed CKD***
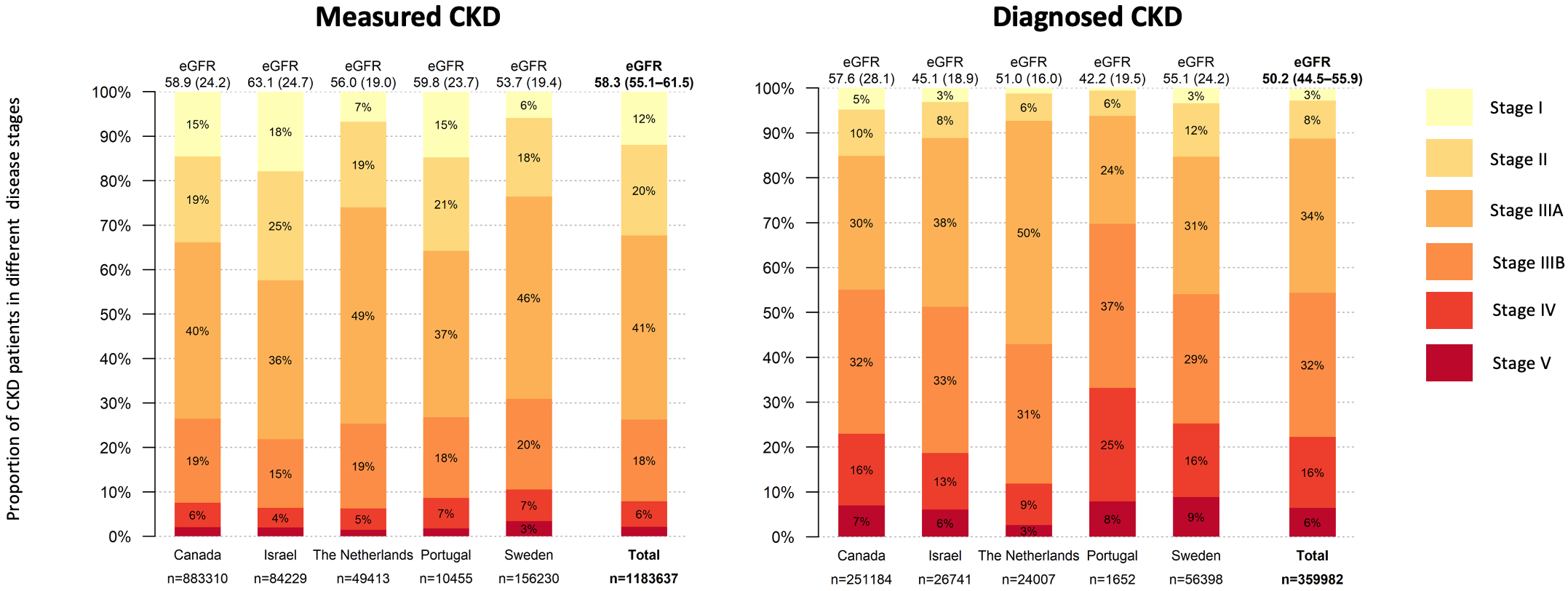


*Measured CKD*, patients with KDIGO confirmed CKD using UACR and eGFR. *Diagnosed CKD*, patients who have a registered CKD diagnosis. Stage I: eGFR ≥90 ml/min/1·73 m^2^ and UACR 30-300 mg/g [3-30 mg/nmol] Stage II: eGFR 60-89 ml/min/1·73 m^2^ and UACR 30-300 mg/g [3-30 mg/nmol] Stage IIIA: eGFR 45-59 ml/min/1·73 m^2^  Stage IIIB: eGFR 30-44 ml/min/1·73 m^2^) Stage IV: eGFR 15-29 ml/min/1·73 m^2^ Stage V: eGFR <15 ml/min/1·73 m^2^ eGFR, estimated glomerular filtration rate, ml/min/1·73 m^2^. UACR, Urine albumin-to-creatinine ration. UK, United Kingdom.

**Supplementary References**

1. Vigen R, Maddox TM, Allen LA. Aging of the United States population: impact on heart failure. Curr Heart Fail Rep. 2012 Dec;9(4):369–74.

2. Kuiper JG, Bakker M, Penning-van Beest FJA, Herings RMC. Existing Data Sources for Clinical Epidemiology: The PHARMO Database Network. Clin Epidemiol. 2020;12:415–22.

3. van Herk-Sukel MPP, van de Poll-Franse LV, Lemmens VEPP, Vreugdenhil G, Pruijt JFM, Coebergh JWW, et al. New opportunities for drug outcomes research in cancer patients: the linkage of the Eindhoven Cancer Registry and the PHARMO Record Linkage System. Eur J Cancer. 2010 Jan;46(2):395–404.

4. WHO Anatomical Therapeutic Chemical Classification System [Internet]. [cited 2021 Dec 22]. Available from: https://www.whocc.no/atc_ddd_index/

5. International Classification of Primary Care [Internet]. [cited 2021 Dec 22]. Available from: https://www.nhg.org/themas/artikelen/icpc

6. Dutch Hospital Data Foundation [Internet]. [cited 2021 Dec 22]. Available from: https://www.dhd.nl/Paginas/home.aspx

7. International Classification of Diseases (ICD) [Internet]. [cited 2021 Dec 22]. Available from: https://www.who.int/standards/classifications/classification-of-diseases

8. Dutch Healthcare Authority declaration codes[Internet]. [cited 2021 Dec 22]. Available from: https://www.opendisdata.nl/downloads

9. Dutch Classification of Procedures [Internet]. [cited 2021 Dec 22]. Available from: https://class.whofic.nl/browser.aspx

10. Norwegian Cause of Death Registry [Internet]. Norwegian Institute of Public Health. [cited 2021 Dec 22]. Available from: https://www.fhi.no/en/hn/health-registries/cause-of-death-registry/

11. Sicras-Mainar A, Enriquez JL, Hernández I, Sicras-Navarro A, Aymerich T, Leon M. PMU146 Validation and representativeness of the Spanish BIG-PAC Database: Integrated computerized medical records for research into epidemiology, medicines and health resourse use (Real world evidence). Value in Health;22:S734. Available from: https://www.sciencedirect.com/science/article/pii/S1098301519341427

12. Norhammar A, Bodegård J, Nyström T, Thuresson M, Eriksson JW, Nathanson D. Incidence, prevalence and mortality of type 2 diabetes requiring glucose-lowering treatment, and associated risks of cardiovascular complications: a nationwide study in Sweden, 2006-2013. Diabetologia. 2016 Aug;59(8):1692–701.

13. Ludvigsson JF, Andersson E, Ekbom A, Feychting M, Kim J-L, Reuterwall C, et al. External review and validation of the Swedish national inpatient register. BMC Public Health. 2011 Jun 9;11:450.

14. Ludvigsson JF, Otterblad-Olausson P, Pettersson BU, Ekbom A. The Swedish personal identity number: possibilities and pitfalls in healthcare and medical research. Eur J Epidemiol. 2009;24(11):659–67.

15. Herrett E, Gallagher AM, Bhaskaran K, Forbes H, Mathur R, van Staa T, et al. Data Resource Profile: Clinical Practice Research Datalink (CPRD). Int J Epidemiol. 2015 Jun;44(3):827–36.

16. Wolf A, Dedman D, Campbell J, Booth H, Lunn D, Chapman J, et al. Data resource profile: Clinical Practice Research Datalink (CPRD) Aurum. Int J Epidemiol. 2019 Dec 1;48(6):1740–1740g.
